# Diversity of nitrogen-fixing rhizobacteria associated with sugarcane: a comprehensive study of plant-microbe interactions for growth enhancement in *Saccharum* spp.

**DOI:** 10.1186/s12870-020-02400-9

**Published:** 2020-05-18

**Authors:** Rajesh Kumar Singh, Pratiksha Singh, Hai-Bi Li, Qi-Qi Song, Dao-Jun Guo, Manoj K. Solanki, Krishan K. Verma, Mukesh K. Malviya, Xiu-Peng Song, Prakash Lakshmanan, Li-Tao Yang, Yang-Rui Li

**Affiliations:** 1 Key Laboratory of Sugarcane Biotechnology and Genetic Improvement (Guangxi), Ministry of Agriculture, Sugarcane Research Center, Chinese Academy of Agricultural Sciences, Guangxi Key Laboratory of Sugarcane Genetic Improvement, Sugarcane Research Institute, Guangxi Academy of Agricultural Sciences, Nanning, 530007 Guangxi China; 2 College of Agriculture, State Key Laboratory of Conservation and Utilization of Subtropical Agro-bio resources, Guangxi University, Nanning, 530005 China; 3 Guangxi Key Laboratory of Crop Genetic Improvement and Biotechnology, Nanning, 530007 Guangxi China; 4 Department of Food Quality and Safety, Institute for Post-harvest and Food Sciences, The Volcani Center, Agricultural Research Organization, 7528809 Rishon LeZion, Israel; 5 Queensland Alliance for Agriculture and Food Innovation, The University of Queensland, St Lucia, QLD 4072 Australia

**Keywords:** Genetic diversity, GFP, Microbe-plant interactions, Nitrogen-fixing bacteria, PGPR, ^15^N_2_ isotope, qRT-PCR, Sugarcane

## Abstract

**Background:**

Nitrogen is an essential element for sugarcane growth and development and is generally applied in the form of urea often much more than at recommended rates, causing serious soil degradation, particularly soil acidification, as well as groundwater and air pollution. In spite of the importance of nitrogen for plant growth, fewer reports are available to understand the application and biological role of N_2_ fixing bacteria to improve N_2_ nutrition in the sugarcane plant.

**Results:**

In this study, a total of 350 different bacterial strains were isolated from rhizospheric soil samples of the sugarcane plants. Out of these, 22 isolates were selected based on plant growth promotion traits, biocontrol, and nitrogenase activity. The presence and activity of the *nifH* gene and the ability of nitrogen-fixation proved that all 22 selected strains have the ability to fix nitrogen. These strains were used to perform 16S rRNA and *rpoB* genes for their identification. The resulted amplicons were sequenced and phylogenetic analysis was constructed. Among the screened strains for nitrogen fixation, CY5 (*Bacillus megaterium*) and CA1 (*Bacillus mycoides*) were the most prominent. These two strains were examined for functional diversity using Biolog phenotyping, which confirmed the consumption of diverse carbon and nitrogen sources and tolerance to low pH and osmotic stress. The inoculated bacterial strains colonized the sugarcane rhizosphere successfully and were mostly located in root and leaf. The expression of the *nifH* gene in both sugarcane varieties (GT11 and GXB9) inoculated with CY5 and CA1 was confirmed. The gene expression studies showed enhanced expression of genes of various enzymes such as catalase, phenylalanine-ammonia-lyase, superoxide dismutase, chitinase and glucanase in bacterial-inoculated sugarcane plants.

**Conclusion:**

The results showed that a substantial number of *Bacillus* isolates have N-fixation and biocontrol property against two sugarcane pathogens *Sporisorium scitamineum* and *Ceratocystis paradoxa*. The increased activity of genes controlling free radical metabolism may at least in part accounts for the increased tolerance to pathogens. Nitrogen-fixation was confirmed in sugarcane inoculated with *B. megaterium* and *B. mycoides* strains using N-balance and ^15^N_2_ isotope dilution in different plant parts of sugarcane. This is the first report of *Bacillus mycoides* as a nitrogen-fixing rhizobacterium in sugarcane.

## Background

Sugarcane (*Saccharum officinarum* L.) is the world’s largest sugar crop and globally the second largest source of biofuel [1, 2]. It is also an increasingly important source of raw materials for animal feed, paper production, and many biomass-based products [3]. China ranks the third-largest sugarcane growing country, producing about ten million tons of sugar annually [2]. Yet, it is now the largest sugar importer in the world due to increasing local consumption. Given this commercial reality, there is a strong impetus to increase sugarcane production area and crop productivity in China. Sugarcane is a fast-growing high-biomass crop and its nutrient and water requirements are relatively large. There is a huge variation for nitrogen (N) fertilizer application for sugarcane production between countries, ranging from as little as 60 kg N ha^− 1^ in some regions of Brazil to as high as 755 kg N ha^− 1^ in some parts of China [4]. In China, the excessive application of N fertilizer in sugarcane crop, spurred by the low cost of fertilizer and as an insurance strategy to achieve high cane yield, is causing considerable soil degradation as well as air and groundwater pollution [5]. Further, the high use of N fertilizer adversely affects sugar quality and dramatically alters soil biota, which often results in a substantial decline in beneficial microflora associated with N mineralization and supply. Compounding these issues is the regulatory pressure felt across agriculture, including the sugarcane industry, to reduce greenhouse gas emission from agriculture to mitigate soil degradation and climate change [6].

The need for reducing chemical N fertilizer dependency prompted research on sustainable alternative nitrogen sources for crop production [7]. Soil micro-organisms and plant microbial endophytes including rhizobacteria have been reported to promote plant growth, suppress pathogens and in some instances improve abiotic stress tolerance [8, 9]. More specifically, plant growth-promoting rhizobacteria (PGPR) with nitrogen-fixing ability are reported to be a valuable source of nitrogen for sustainable crop production as well as to maintain soil fertility [3]. Several groups of soil and root-associated nitrogen-fixing microorganisms such as *Azotobacter vinelandii* [10], *Azospirillum brasilense, Azospirillum zeae,* and *Pseudomonas stutzeri* [11], *Acetobacter diazotrophicus* [12], *Achromobacter insolitus* [13], *Bacillus megaterium* [14], *Bacillus rhizosphaerae* [15], *Burkholderia tropica* [16], *Burkholderia xenovorans* [17], *Burkholderia silvatlantica* [18], and *Burkholderia caballeronis* [19], *Bradyrhizobium. japonicum* and *B. elkanii*, [20], *Delftia tsuruhatensis* [21], *Enterobacter sacchari* [22], *Gluconacetobacter diazotrophicus* [23], *Gluconacetobacter diazotrophicus* [24], *Stenotrophomonas maltophilia* [25], *Pseudomonas koreensis*, and *P. entomophila* [7] have been found to colonize different crops and stimulate plant growth either directly or indirectly. Their activity, however, is influenced by crop species, soil type and soil condition [26]. Therefore, it is important to isolate, identify, and culture PGPRs associated with sugarcane that functions optimally in different soil types and climatic conditions to promote crop growth and yield.

The nitrogen-fixing *Bacillus* species have the potential to use as a microbial bio-fertilizer and biocontrol in agriculture sectors [27]. Therefore, in this study, we focused on the bacteria belonging to nitrogen-fixing *Bacillus* species. *Bacillus* spp. occur in very different soil types and they can be cultured. They have a complex cell wall and, stress-resistant endospore they produce antibiotics and extracellular lytic enzymes, and tolerate adverse environmental conditions for extended periods [28, 29]. Several *Bacillus* species such as *Bacillus tequilensis*, *B. megaterium*, *B. cereus* [30, 31], *B. amyloliquefaciens*, *B. aryabhattai*, *B. safensis*, *B. aerophilus*, *B. subtilis* [31, 32], *B. rhizosphaerae* [15], *B. pumilus* [31], *B. fluminensis* [33] and *B. indica* [34] have been isolated from sugarcane. These micro-organisms promote plant growth directly via nitrogen fixation, phosphate solubilization and production of phytohormones, and indirectly through the production of antibiotics, hydrolytic enzymes and siderophores [3, 34–37].

Many *Bacillus* species fix N_2_ and the occurrence of N_2_ fixing bacteria in sugarcane was first reported by Dobereiner and Ruschel [38], which was confirmed by later studies [23, 39, 40]. Based on nitrogenase activity, Xie et al. [41] showed that *Bacillus brevis, B. cereus, B. circulans, B. firmus, B. licheniformis, B. megaterium, B. pumilus, and B. subtilis* associated with rice have the capacity to fix nitrogen. Recently, *Paenibacillus odorifer, P. graminis, P. peoriae,* and *P. brasilensis* have been described as nitrogen fixers in other plants [42–44], with the presence of *nifH* gene established in*, P. graminis* and *P. odorifer* [43, 45].

Microbial colonization is an important aspect of successful plant-microorganism interactions [46]. In many instances, artificially inoculated PGPRs failed to colonize target hosts grown in the soil. The reason for poor colonization by externally supplied PGPRs, especially when plants are grown in soil, is not known, and this currently limits the application of PGPRs in many commercial crops, including sugarcane [47]. Hence, the objectives of this study are (i) to isolate nitrogen-fixing microorganisms from the rhizosphere of Chinese sugarcane germplasm and characterize them for nitrogen fixation, plant growth promotion and biocontrol of sugarcane pathogens, and (ii) to understand how the host and the growing environment control the colonization process. Using several experimental tools and strategies relevant to rhizosphere and microbiome association studies, such as the expression of the *nifH* gene, ^15^N_2_ tracer studies, confocal microscopy, and N_2_-fixation-associated metabolic changes, this important plant-microbe interaction has been studied and the results are presented here.

## Results

### Isolation and characterization of rhizosphere bacteria with PGP ability

**Fig. 1:**
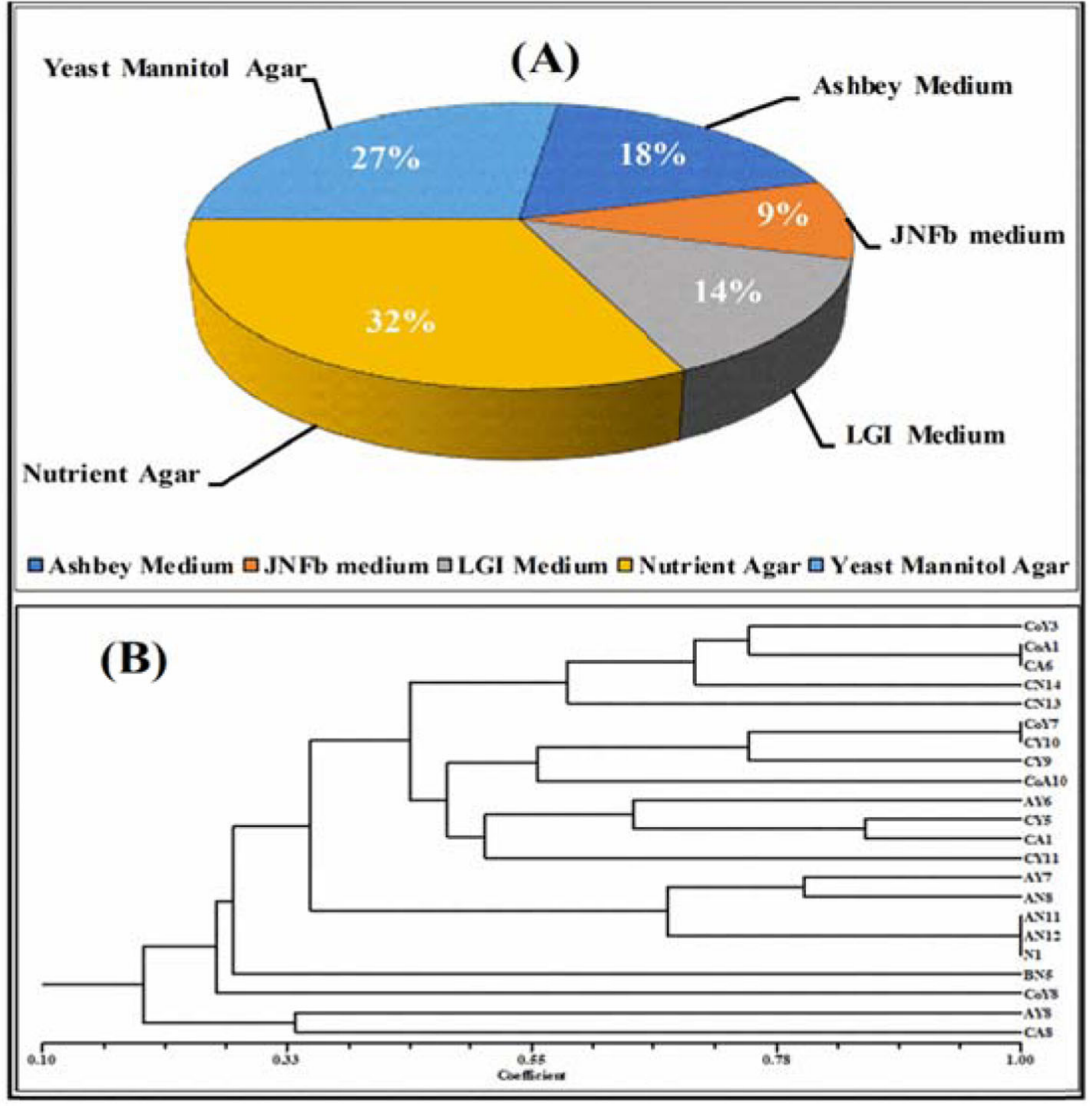
**a** Assortment of nitrogen-fixing micro-organisms from the rhizosphere of sugarcane plants in a different medium, **b** A dendrogram was constructed on the basis of functional characterization of different PGP traits for all selected isolates

A total of 350 bacterial isolates were obtained from the sugarcane rhizosphere. Out of these, 102 isolates were selected with different PGP traits, and nitrogenase activity and were tested in vitro for antagonistic activity against sugarcane pathogens. Following this screening, 22 of them were selected for further studies (Fig. 1a-b).

All the isolated sugarcane rhizosphere bacteria were primarily screened to analyze their biocontrol property against the pathogens *Sporisorium scitamineum* and *Ceratocystis paradoxa*. A total of 18 such isolates with biocontrol property were selected. The data in Table 1, indicated that about 40 (9) and 60% (13) of the isolates were antagonistic to *S. scitamineum* and *C. paradoxa* respectively. All the selected isolates were screened for their capacity to solubilize phosphate using Pikovskaya’s plates. The results showed that 82% (18) of tested isolates produced a halo zone, indicating their capacity to produce organic acids to solubilize the tri-calcium phosphate in the media. Among the 22 bacterial isolates tested, 45.5% (10) were able to produce an orange halo zone on the chrome azurol S agar medium indicating siderophores production. Further, about 60% (13) of the isolates produced ammonia and 18% (4) produced hydrogen cyanide (HCN) (Table 1).

**Table 1:** In vitro characterization of rhizospheric bacteria on the basis of plant growth promotion attributes exhibited by selected isolates

Culture Code	Phosphate	Siderophore	Ammonia	HCN	ACC deaminase	Antifungal activity	
						***Sporisorium scitamineum***	***Ceratocystis paradoxa***
CoY3	+	–	+++	–	–	+	+
CoY7	+	–	++	–	+	++	–
CoY8	–	–	++	+	–	–	++
CoA1	+	–	++	–	–	–	+
CoA10	+++	–	–	–	–	++	–
AY6	++	+++	–	++	+	++	–
AY7	+++	++	–	–	+	–	+++
AY8	–	++	++	–	–	–	–
AN8	+++	+++	–	++	+	–	+
AN11	++	+++	–	–	–	–	+++
AN12	++	+++	–	–	–	–	++
BN5	++	–	–	–	–	–	–
CY5	++	++	++	–	+	++	++
CY9	++	–	++	–	–	++	–
CY10	++	–	++	–	+	++	–
CY11	–	–	++	–	+	+	+
CA1	++	+++	++	+++	+	++	+
CA6	++	–	+++	–	–	–	+
CA8	–	+++	–	–	+	–	–
CN13	++	–	++	–	–	–	–
CN14	++	–	++	–	+	–	+
N1	++	++	–	–	–	–	++

(**+**) = low activity; (**++**) = moderate activity; (**+++**) = strong activity; (−) = no activity

Phosphate and siderophore activity of the bacterial isolates 3 mm or greater clear zone of inhibitions on suitable medium after 3–5 days of incubation at 30 ± 2 °C.

Antifungal activity by dual culture plate measured as a zone of inhibition after 3–5 days of incubation at 26 ± 2 °C.

The ability to synthesize Indole-3-acetic acid (IAA) is an important feature of PGPR isolates. The data in Table 2, shows that the isolates had a very diverse capacity to synthesize IAA. These variations ranged from 11.42 ± 0.49 to 44.88 ± 0.19 μ g mL^− 1^ in a medium without tryptophan but the addition of tryptophan resulted in 29.65 ± 0.61 to 316.84 ± 2.5 μ g mL^− 1^. The isolates CoY8 and CY5 showed the lowest and highest IAA production, respectively in the absence of tryptophan. However, in the presence of tryptophan, the lowest and highest production of IAA was observed in isolates CA8 and AY8, respectively. The level of nitrogenase activities varied greatly amongst the twenty-two bacterial isolates tested. The nitrogen-fixing ability among the isolates ranged from 2.40 ± 0.24 to 26.59 ± 2.0 n moL C_2_H_4_ mg protein h^− 1^. Isolate AN11 showed the highest and CY10 recorded the lowest activity using an acetylene reduction assay. 1-aminocyclopropane-1-carboxylate (ACC) deaminase activity was determined as the ability to use ACC as the sole N_2_ source. Of the 22 isolates, 10 (45.5%) were able to grow normally on the DF medium supplemented with 3 m moL L^− 1^ of ACC after 36–48 h incubation at 30 ± 2 °C. Subsequently, the color of bacteria appeared dark in the DF medium with ACC. On the basis of the above results, 10 of the isolates were selected for further quantitative tests and found varying levels of ACC deaminase amongst them (Table 2). The highest activity was found in the CY5 (75.63 ± 3.35) with CA8 registering the lowest level (16.47 ± 0.42), and all these selected strains showed the amplification approximately 755 bp of *acdS* gene (Additional file 1: Fig. S1).

**Table 2:** In vitro screening of the bacterial isolates for IAA production, ARA, and ACC deaminase activity

Culture Code	IAA (μ g mL^**− 1**^)		ARA (n moL C_**2**_H_**4**_ mg protein h^**− 1**^)	ACC (nmol α-ketobutyrate mg^**− 1**^ h^**− 1**^)
	A-Tryptophan	P-Tryptophan		
CoY3	11.61 ± 0.49^l^	36.35 ± 0.10^nm^	10.04 ± 1.65^h^	–
CoY7	15.70 ± 0.34^h^	44.82 ± 0.61^k^	16.65 ± 1.25^fg^	34.72 ± 5.33^d^
CoY8	11.42 ± 0.49^l^	49.26 ± 0.69^j^	17.65 ± 0.27^ef^	–
CoA1	17.12 ± 0.33^fg^	42.14 ± 0.21^l^	5.53 ± 0.56^i^	–
CoA10	12.56 ± 0.25^kl^	51.84 ± 0.10^ij^	16.74 ± 0.18^fg^	–
AY6	14.10 ± 0.34^ij^	49.20 ± 0.34^j^	21.38 ± 2.42^cd^	19.60 ± 0.89^e^
AY7	26.25 ± 0.51^d^	53.84 ± 0.27^i^	21.59 ± 3.65^cd^	39.80 ± 1.10^c^
AY8	17.27 ± 0.20^fg^	316.84 ± 2.5^b^	25.89 ± 2.08^ab^	–
AN8	19.13 ± 0.51^e^	290.57 ± 2.62^c^	24.73 ± 0.61^ab^	17.42 ± 0.55^e^
AN11	39.92 ± 2.95^b^	104.62 ± 0.51^h^	26.59 ± 2.05^a^	–
AN12	17.37 ± 0.61^f^	112.27 ± 1.28^g^	19.70 ± 0.67^de^	–
BN5	15.41 ± 0.11^h^	271.06 ± 2.45^d^	23.62 ± 1.15^bc^	–
CY5	44.88 ± 0.19^a^	35.01 ± 0.30^mn^	23.87 ± 2.55^bc^	75.63 ± 3.35^a^
CY9	14.03 ± 0.30^ij^	49.33 ± 0.63^j^	25.87 ± 1.72^ab^	–
CY10	14.98 ± 0.26^hi^	33.84 ± 0.11^n^	2.40 ± 0.24^j^	32.92 ± 4.23^d^
CY11	43.77 ± 0.23^a^	175.73 ± 0.91^e^	16.12 ± 0.94^fg^	41.57 ± 3.91^c^
CA1	16.14 ± 0.10^gh^	130.96 ± 1.81^f^	15.94 ± 2.11^fg^	49.61 ± 1.15^b^
CA6	13.28 ± 0.25^jk^	37.10 ± 0.41^m^	14.23 ± 1.07^g^	–
CA8	17.42 ± 0.18^f^	29.65 ± 0.61^o^	15.08 ± 1.17^fg^	16.47 ± 0.42^e^
CN13	28.28 ± 0.47^c^	36.12 ± 1.93^mn^	16.87 ± 0.92^fg^	21.05 ± 1.04^e^
CN14	13.90 ± 0.19^ij^	37.56 ± 0.30^m^	16.92 ± 1.71^fg^	–
N1	18.64 ± 0.15^e^	339.07 ± 5.56^a^	17.69 ± 0.76^ef^	–
SEM	0.39	0.89	0.86	1.62
CD (*P* = 0.05)	1.12	2.55	2.44	4.87
CV (%)	3.40	1.50	8.30	7.60

*A-Tryptophan* absence of tryptophan, *P-Tryptophan* presence of tryptophan.

Means followed by the same letter within a row are not significantly different (*p* ≤ 0.05) according to Duncan’s Multiple Range Test (DMRT). *SEM* standard error of the difference between means, *CD* critical difference, *CV* coefficient of variation

### Molecular characterization of bacterial isolates

In the present study, the 16S and *rpoB* rRNA gene amplification was done. The amplified fragment was used for 16S rRNA gene partial sequencing and *basic local alignment search tool* (BLAST) analysis for judging the sequence similarity with the national center for biotechnology information (NCBI) GenBank database. The results displayed that all the isolates belonged to the *Bacillus* genus (Table 3). Based on the similarity value ≥97% score, we separated the *Bacillus* into 13 different species including 3 belongs to *B.* species, 1 to *B. licheniformis*, 3 to *B. pumilus*, 2 to *B. safensis*, 1 to *Firmicutes bacterium*, 1 to *B. luciferensis*, 1 to *Paenibacillus lautus*, 2 to *B. cereus*, 2 to *B. subtilis*, 3 to *B. thuringiensis*, 1 to *B. megaterium*, 1 to *B. aryabhattai*, and 1 to *B. mycoides*. In addition, we found that some isolates belonged to the same species, as determined by the 16S rRNA gene sequences. Another primer set of *rpoB* gene was also used for the amplification of partial sequences of the genes. As shown in Table 3, the *rpoB* gene sequence homology analysis failed to discriminate against the isolates CoA10, AY6, and CY11.

**Table 3:** Identification of selected bacterial strains isolated from sugarcane-based on 16S rRNA and *rpoB* gene sequence

Isolates	Identified by two genes: A (16S rRNA) and B *(rpoB)*		% identity^**a**^		No. of Nucleotides^**b**^		Accessions Number^**c**^	
	A	B	A	B	A	B	A	B
CoY3	*Bacillus* spp.	*Bacillus* spp.	98	97	1452	779	KY652111	KY652133
CoY7	*Bacillus licheniformis*	*Bacillus licheniformis*	98	96	1396	547	KY652112	KY652134
CoY8	*Bacillus pumilus*	*Bacillus pumilus*	99	99	1447	1166	KY652113	KY652135
CoA1	*Bacillus safensis*	*Bacillus safensis*	99	99	1447	1178	KY652114	KY652136
CoA10	*Firmicutes bacterium*	*Bacillus cereus*	98	98	1490	1080	KY652115	KY652137
AY6	*Bacillus luciferensis*	*Bacillus amyloliquifensis*	98	96	1497	581	KY652116	KY652138
AY7	*Paenibacillus lautus*	*Paenibacillus lautus*	97	96	1389	352	KY652117	KY652139
AY8	*Bacillus cereus*	*Bacillus cereus*	99	99	1414	1104	KY652118	KY652140
AN8	*Bacillus subtilis*	*Bacillus subtilis*	99	99	1462	1149	KY652119	KY652141
AN11	*Bacillus thuringiensis*	*Bacillus thuringiensis*	98	96	1474	778	KY652120	KY652142
AN12	*Bacillus subtilis*	*Bacillus subtilis*	99	97	1458	1094	KY652121	KY652143
BN5	*Bacillus cereus*	*Bacillus cereus*	98	96	1392	840	KY652122	KY652144
CY5	*Bacillus megaterium*	*Bacillus megaterium*	98	97	1403	690	KY652123	KY652145
CY9	*Bacillus safensis*	*Bacillus safensis*	99	98	1354	1164	KY652124	KY652146
CY10	*Bacillus pumilus*	*Bacillus pumilus*	99	98	1375	1165	KY652125	KY652147
CY11	*Bacillus aryabhattai*	*Bacillus altitudinis*	99	98	1455	1139	KY652126	KY652148
CA1	*Bacillus mycoides*	*Bacillus mycoides*	98	96	1413	781	KY652127	KY652149
CA6	*Bacillus pumilus*	*Bacillus pumilus*	99	98	1459	1155	KY652128	KY652150
CA8	*Bacillus thuringiensis*	*Bacillus thuringiensis*	99	98	1461	1211	KY652129	KY652151
CN13	*Bacillus* spp.	*Bacillus* spp.	97	97	1457	800	KY652130	KY652152
CN14	*Bacillus* spp.	*Bacillus* spp.	98	98	1518	775	KY652131	KY652153
N1	*Bacillus thuringiensis*	*Bacillus thuringiensis*	98	96	1411	694	KY652132	KY652154

^a^The percentage identity with the 16S rDNA/ *rpoB* gene sequence of the closest phylogenetic relative

^b^The number of 16S rDNA/ *rpoB* gene nucleotides used for the alignment

^c^NCBI GenBank accession number of 16S rDNA/ *rpoB* gene

### Phylogenetic structure of 16S rRNA and *rpoB* genes

**Fig. 2:**
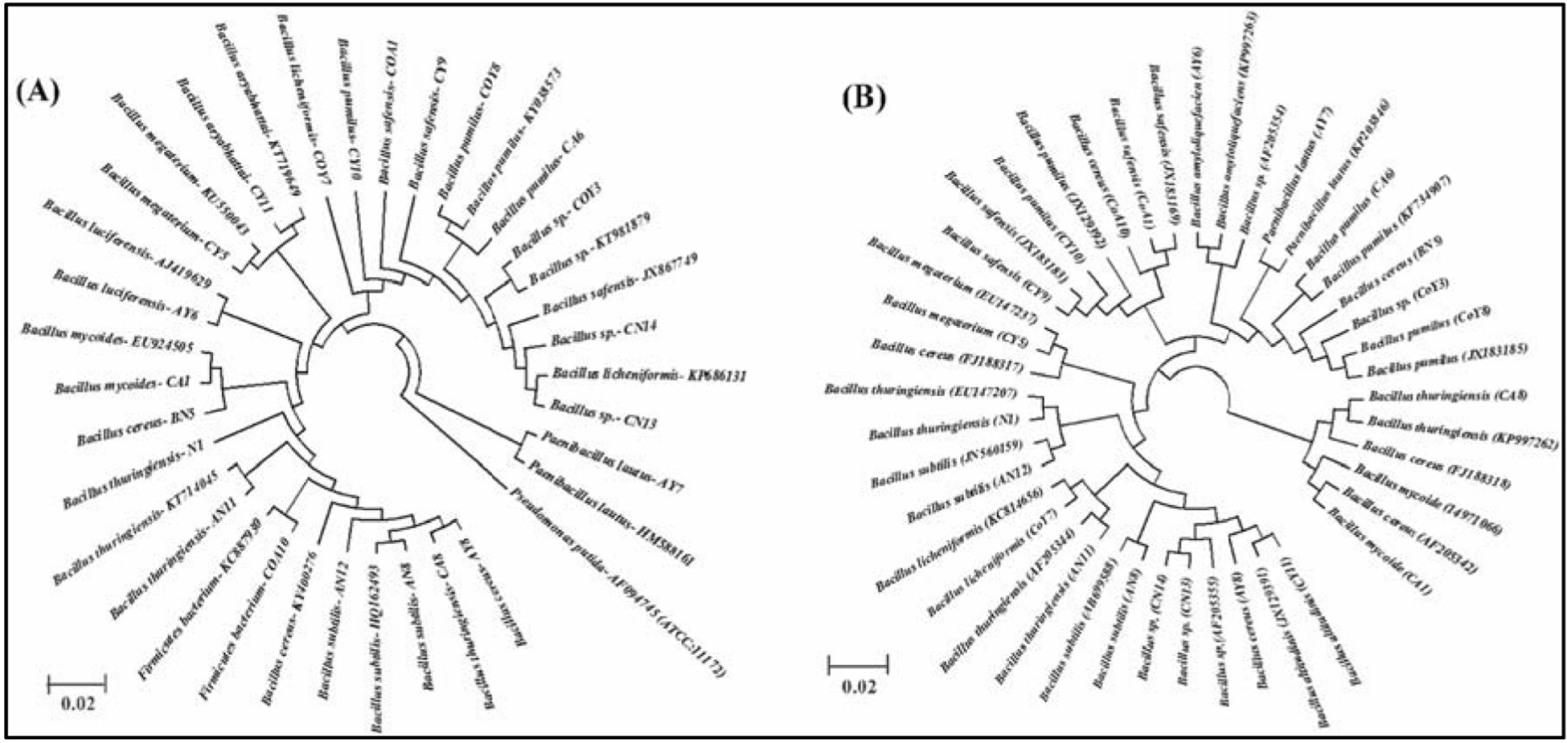
**a-b** Phylogenetic tree based on the partial 16S rRNA and *rpoB* gene sequences of twenty-two potent plant-growth-promoting and nitrogen-fixing *Bacillus* strains screened from sugarcane. Evolutionary distances were calculated using the UPGMA method. Based on bootstrap analysis of 1000 replications are indicated as percent confidence values for particular branching. Scale bar represents the number of changes per base position and *Pseudomonas putida* was used as an outgroup

The individual analysis for a phylogenetic tree for the evolutionary relationship was done. The 16S rRNA and *rpoB* gene partial sequences of the isolated strains were compared with the reference strains of the NCBI GenBank public database. In 16S rRNA genes two major and two minor groups were formed based on the NJ method with 1000 bootstrap sampling (Fig. 2a). *Pseudomonas putida* was used as the reference to separate *Bacillus* strains. However, in the case of the *rpoB* gene three major and two minor groups were formed (Fig. 2b).

### Amplification of the *nifH* gene

To investigate the *nifH* gene for all the selected isolates, the genomic DNA extracted and used to detect the PCR products with an accurate band size of about 360 bp. Results showed that six strains were positive for *nifH* gene amplification (Additional file 2: Fig. S2). The positive isolates were used to establish *nifH* clone libraries and ten clones were selected from each isolate for sequencing. All the sequenced clones were found to be similar to the *nifH* gene by the BlastN search program from the NCBI GenBank database. These isolates were similar to *B. cereus* (AY8), *B. subtilis* (AN8), *B. cereus* (BN5), *B. megaterium* (CY5), *B. pumilus* (CY10), and *B. pumilus* (CA6). The *nifH* sequences identified were submitted to NCBI GenBank and their accession numbers are from KY652155 to KY652160.

### Characterization of genomic fingerprinting

**Fig. 3:**
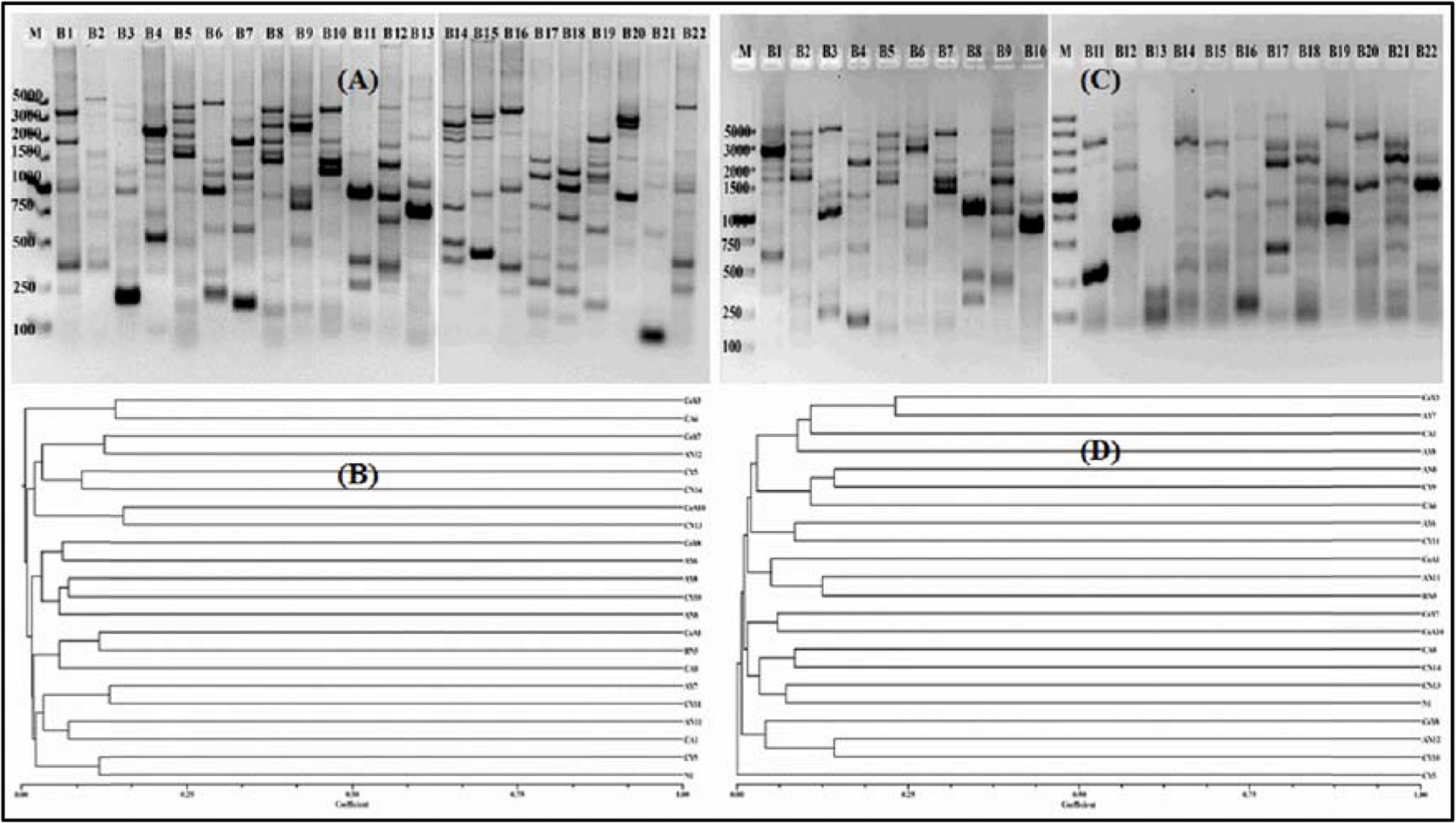
Genotypic fingerprinting of the screened bacterial isolates from the sugarcane rhizosphere obtained by using genomic DNA. Banding patterns for BOX and ERIC-PCR are shown in **a** and **c**. White space represents the joining of two gels. The dendrogram (**b** and **d**) was obtained from the similarity coefficient and clustering was done by using the UPGMA algorithm with the NTSYS software program. Strain Codes: B1. CoY3, B2. CoY7, B3. CoY8, B4. CoA1, B5. CoA10, B6. AY6, B7. AY7, B8. AY8, B9. AN8, B10. AN11, B11. AN12, B12. BN5, B13. CY5, B14. CY9, B15. CY10, B16. CY11, B17. CA1, B18. CA6, B19. CA8, B20. CN13, B21. CN14 and B22. N1. M, molecular size marker from 100 bp–5 kb (Takara)

A genomic fingerprint was examined by A1R-based repetitive extragenic palindromic (BOX) and enterobacterial repetitive intergenic consensus (ERIC) PCR using the purified DNA of selected isolates from the sugarcane rhizosphere. Many polymorphic bands were observed approximately ranging between 100 bp and about 5 kb. The genomic DNA fingerprints generated from all the isolates were clearly distinguished from each other. High-quality fingerprint profiles were visualized on agarose gels with each primer set (Fig. 3). The BOX and ERIC-PCR fingerprints results were very complicated with several polymorphic bands with different intensity. A total of 174 bands ranging from 50 bp to 5 kb were generated from all the 22 selected *Bacillus* strains through BOX PCR. The isolate B9 and B14 showed the maximum 11 bands, after this B1, B4, and B7 showed a similar number of bands (10), while a minimum of 4 bands was detected in B2, and B4 isolates (Fig. 3a; Additional file 3: Fig. S3). For ERIC-PCR the faint bands were frequently observed and approximately 50 bp to 5 kb of 146 bands were identified. The maximum number of bands was observed in three strains i.e., B1, B2, and B9 (10), whereas the minimum bands were found in B13 (3) (Fig. 3c; Additional file 3: Fig. S4).

BOX-PCR fingerprints showed more genotypic diversity for all selected *Bacillus* spp. as compared to ERIC fingerprints, and a dendrogram was created of fingerprint bands to generate their relatedness (Fig. 3). The data were examined by Jaccard similarity coefficients and the neighbor-joining method based on UPGMA. Two major clusters, one comprising two isolates and the other comprising twenty isolates were found in dendrogram created through BOX PCR fingerprints (Fig. 3b). These eighteen isolates were further divided into two sub-groups, one contained 6 isolates and the other had fourteen isolates. In the ERIC-PCR, all the twenty-two isolates showed two major clusters, containing one and twenty-one isolates, respectively. The twenty-one isolates were divided into two sub-groups, which contained 3 and 17 isolates, and all were grouped into different clusters (Fig. 3d).

### Biolog phenotypic profiling of CY5 and CA1

**Fig. 4:**
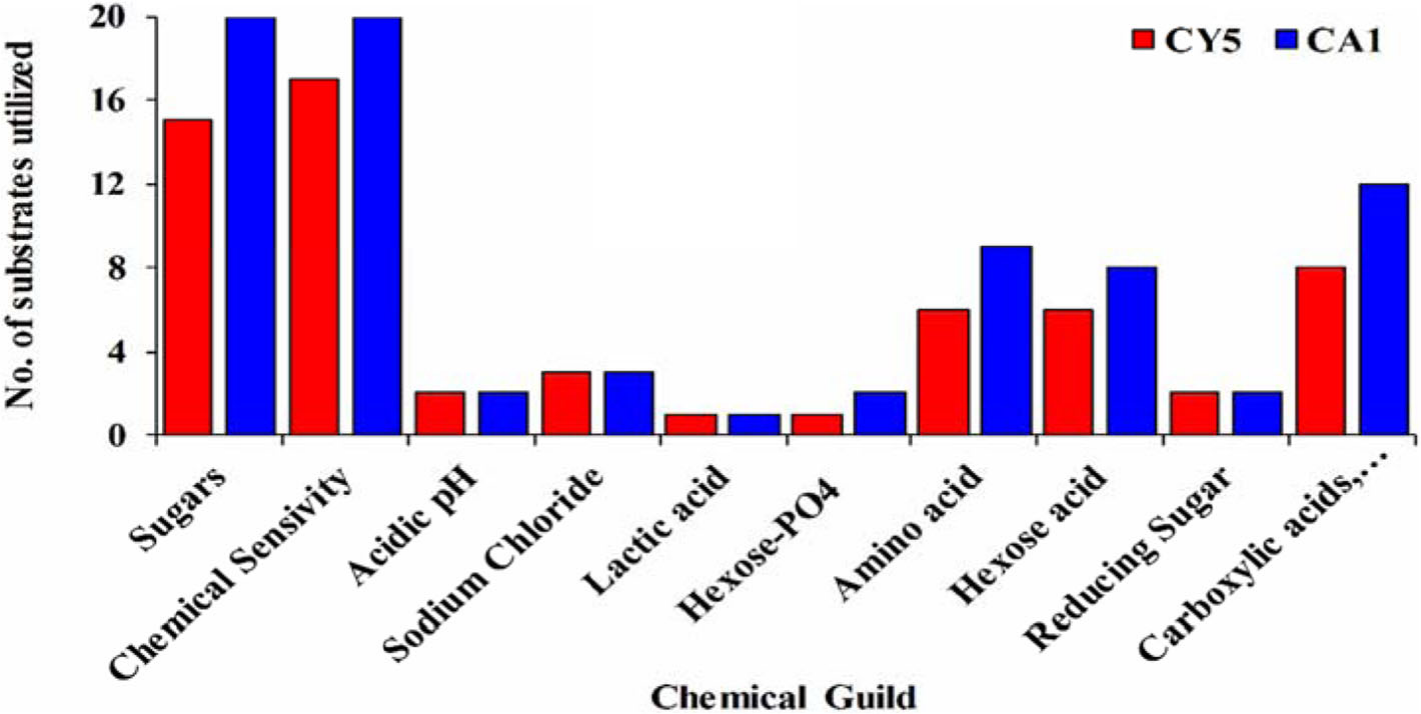
Pattern of phenotypic profiles in CY5 and CA1 isolates in the presence of different carbon substrate utilization using BIOLOG Phenotype Micro-Array™ plates GNIII

The substrate utilization patterns for the selected strains (CY5 and CA1) were established for metabolic potential with numerous groupings such as carbon (C), and nitrogen (N) sources, tolerance of osmotic stress and metabolic activity over a wide range of pH (3.5–10). The physiological, biochemical, and chemical sensitivity of the isolates was performed by the Biolog system based on substrate use. The results showed that the strain CA1 utilized more carbon sources as compared to CY5 i.e. sugars (74.07%), carboxylic acids (66.67%), hexose acids (88.89%), and amino acids (100.00%), and the highest chemical sensitivity (86.97%) (Additional file 4: Table S1). Along with, reducing sugar, sodium chloride, amino acid, lactic acid, and hexose-PO_4_ were used. In both tested strains, 79 (82.29%) of CA1 and 61 (63.54%) of CY5 compounds were utilized (Fig. 4).

Different nitrogen sources support the growth of CY5 and CA1, indicating that these isolates metabolize compounds such as ammonia, nitrite, nitrate, urea, biuret, l-alanine, glycine, hydroxylamine methylamine, ethylamine, ethanolamine, adenine, cytosine, thymine, uracil, and uric acid, and they might tolerate nearly 95 different forms of nitrogen and some related chemicals even at high concentrations. The tolerance levels of isolates CY5 and CA1 to different concentrations of NaCl (1–10%), potassium chloride (3–6%), sodium sulfate (2–5%), ethylene glycol (5–20%), sodium formate (1–6%), urea (2–7%), sodium lactate (1–12%), sodium phosphate pH 7 (20–200 mM), sodium benzoate pH 5.2 (20–200 mM), ammonium sulfate pH 8 (10–100 mM), sodium nitrate (10–100 mM) and sodium nitrite (10–100 mM) were high. In the case of metabolic activity, both isolates could grow in the pH range from 3.5 to 10. The diversity measures can be used to evaluate the diversity of resources used by micro-organisms indicating their tolerance to a wide range of environments. The diversity parameters defined on the basis of data obtained through Biolog analysis are presented in Table 4.

**Table 4:** Substrate richness diversity parameters calculated for nitrogen, osmolytes and pH through Biolog for selected strains CY5 (*Bacillus megaterium*) and CA1 (*Bacillus mycoides*)

Diversity parameters	Diversity indices measurement through different Biolog Micro-Plates					
	CY5			CA1		
	Nitrogen (PM3B)	Osmolytes (PM9)	pH (PM10)	Nitrogen (PM3B)	Osmolytes (PM9)	pH (PM10)
Dominance_D	0.02512	0.01533	0.01131	0.01551	0.01489	0.01183
Simpson_1-D	0.9749	0.9847	0.9887	0.9845	0.9851	0.9882
Shannon_H	4.176	4.247	4.497	4.319	4.297	4.467
Evenness_e^H/S	0.6783	0.7279	9.35E-01	7.82E-01	7.66E-01	0.9073
Brillouin	0.6053	2.635	2.85	0.8634	2.099	2.291
Menhinick	21.48	11.63	9.399	11.91	8.721	8.018
Margalef	53.02	23.4	21.27	29.89	21.5	20.63
Equitability_J	0.915	0.9304	0.9852	0.9462	0.9415	0.9787
Fisher_alpha	0.0	0.0	585.2	0.0	213.9	127.2
Berger-Parker	0.05006	0.01467	0.009586	0.0154	0.01651	0.01395

**Fig. 5:**
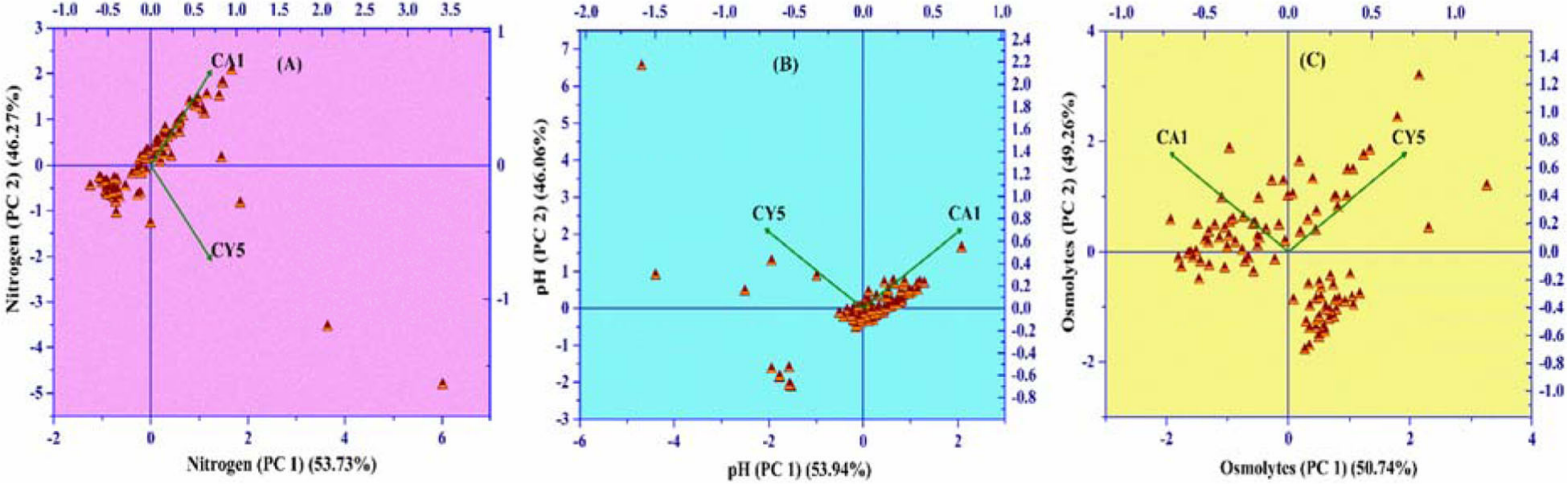
Graphs of principle component analysis results for *Bacillus megaterium* (CY5) and *Bacillus mycoides* (CA1) strains on the basis of BIOLOG^(R)^ micro-plates profile i.e., **a** nitrogen, **b** pH, and **c** osmolytes obtained under the different treatments of phenotypic attributes

A PCA was performed by using PM3B, PM9, and PM10 for a qualitative analysis of the metabolic diversity of both isolates based on the PC score values obtained. Based on the relationships between different substrates, metabolites and strain utilization, the grouping was performed for each individual component. And, for these studies, a separate evaluation was performed for nitrogen source, osmolytes, and pH. The results obtained from nitrogen source (Fig. 5) clarified the metabolic variability of 53.52 and 46.48% according to the first and second PC values, respectively. A figure for PCA scatters plot of osmolytes (Os) showed the first component of PC analysis accounted for 50.74% of the total variance and the second 49.26%. Whereas, in the case of pH, 53.95 and 46.06% of the variance were observed in PC1 and PC2, respectively (Fig. 5a-c).

### Colonization pattern of GFP-tagged CY5 and CA1 on sugarcane

**Fig. 6:**
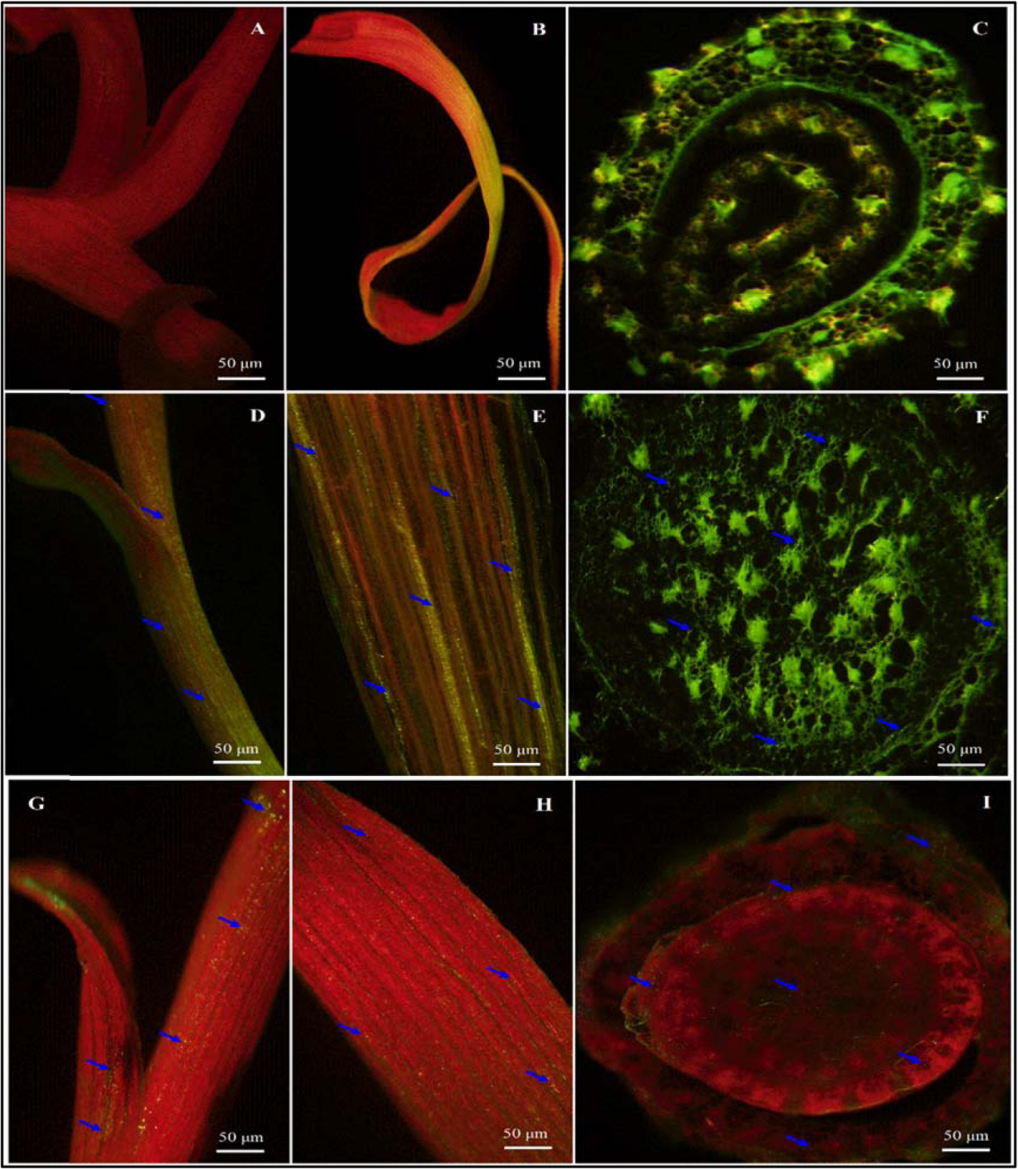
Fluorescence (GFP) micrographs of sugarcane plant colonized by GFP-tagged *Bacillus megaterium* (CY5) and *Bacillus mycoides* (CA1) leaf, stem, and the root of micropropagated plantlets of sugarcane variety GT11. Confocal laser scanning microscopic images present bacterial in green and red dots of auto-fluorescence in everywhere of plant parts respectively. (A-C) Control sugarcane plant parts and (D-I) Sugarcane plant parts inoculated with GFP tagged bacterial strains at 500–530 nm. On the surface of roots, junction area, around the whole root, stem, and leaf. Bacterial cells are indicated with blue arrowheads in single or clustered of bacteria. D-F represent CY5, and G-I represents CA1 strain with bar 50 μm

Two isolates selected from the sugarcane rhizosphere, *B. megaterium* (CY5), and *B. mycoides* (CA1), were used for localization studies using Confocal Laser Scanning Microscopy (CLSM). This technique helped to study plant-microorganism interactions of selected isolates. In this study, both CY5 and CA1 conferred protection against sugarcane pathogens (*S. scitamineum*, and *C. paradoxa*) produced different PGP traits and showed nitrogenase activity in medium (Tables 1 & 2). The data in Fig. 6 showed bacterial cells occupying internal plant tissue. These isolates after 72 h of incubation with GFP pPROBEpTet^r^-TT showed higher fluorescence, and the increased bacterial cell density could be easily detected under CLSM. The control plants without inoculated isolates showed no fluorescent cells (Fig. 6a-c). However, in the sugarcane stem, the transverse sections of roots and in the mesophyll cells of the leaves intercellular bacterial colonization of single or multiple species of bacteria could be seen in the inoculated plant (Fig. 6d-i). In the leaves, the GFP-tagged isolate was for colonization in roots, mostly root hair zones were overshadowed by the green fluorescence produced from xylem vessels, endodermal junction sites, and cell walls. The CLSM images of root, leaf, and stem showed similar colonization patterns and abundance of both CY5 and CA1.

### *nifH* gene expression determined by qRT-PCR in pots under the greenhouse

**Fig. 7:**
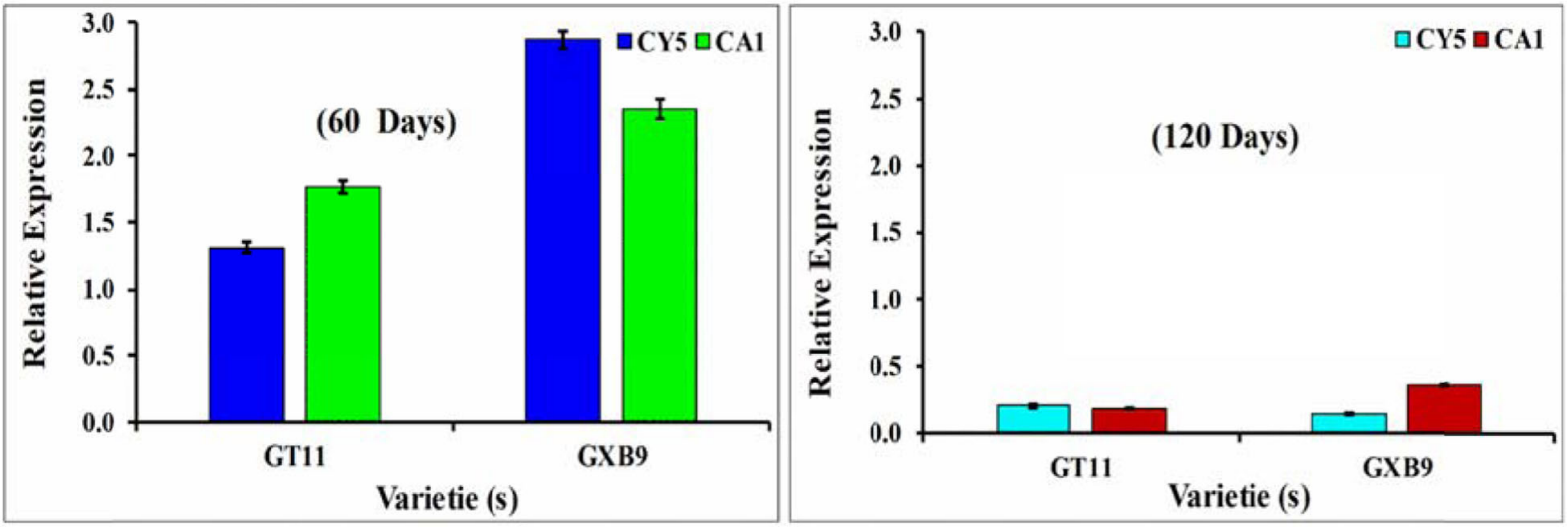
Analysis of inoculated potent strains *Bacillus megaterium* (CY5) and *Bacillus mycoides* (CA1) on the *nifH* expression patterns in two sugarcane varieties (GT11 and GXB9) at 60 and 120 days through qRT-PCR. Data were normalized to the GAPDH expression level. All the data points are the means ± SE (*n* = 3)

The expression of a *nifH* gene by the selected diazotroph bacterial isolates in sugarcane varieties GT11 and GXB9 was monitored by qRT-PCR (Fig. 7). The results from the leaf tissue of inoculated GXB9 plants showed the highest expression of a *nifH* gene on day 60 post-inoculated in both CY5 and CA1 (Fig. 7a), whereas the expression level was higher in GXB9 inoculated with CA1 on day 120 (Fig. 7b). In these studies, *nifH* gene expression was positively detected following the inoculation of both bacterial isolates in both sugarcane varieties at different time intervals.

### Genes expression analysis of CAT, PAL, SOD, CHI, and GLU

**Fig. 8:**
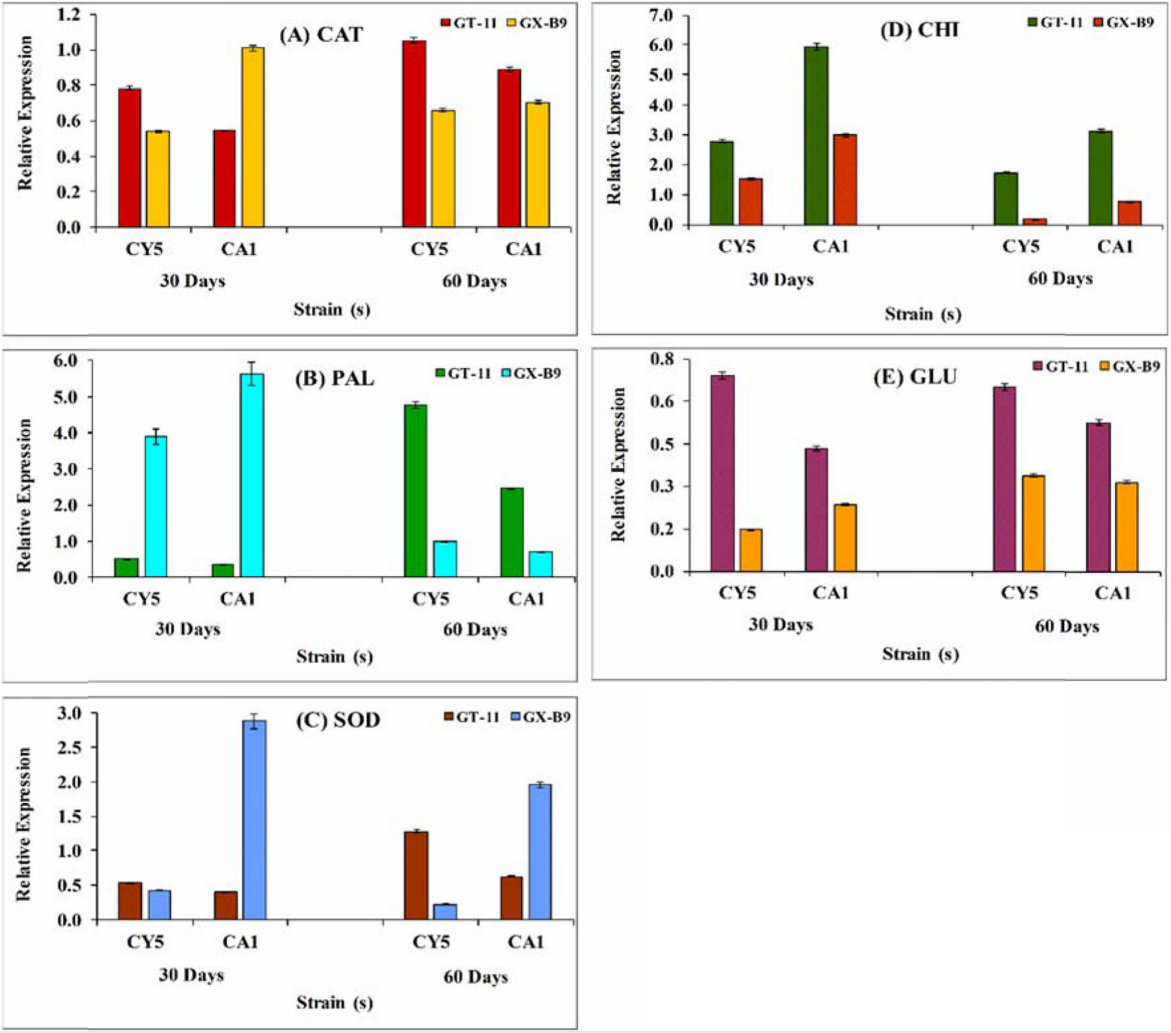
qRT-PCR analysis of differentially expressed genes in leaf tissue of sugarcane varieties GT11 and GXB9 during selected nitrogen-fixing strains inoculation. (A) Catalase (*SuCAT*), (B) Phenylalanine ammonia-lyase (*SuPAL*), (C) Superoxide dismutase (*SuSOD*), (D) Chitinase (*ScCHI*), and (E) Glucanase (*ScGluD1*). Data were normalized to the GAPDH expression level. All data points are the mean ± SE (n = 3)

The differential gene expression of catalase (CAT), phenylalanine ammonia-lyase (PAL), superoxide dismutase (SOD), chitinase (CHI) and glucanase (GLU) in leaf tissues at different stages of sugarcane growth was studied by qRT-PCR (Fig. 8). The data showed a significant change in the expression level of all selected five genes in both sugarcane varieties after CA1 and CY5 inoculation. The expression level of CAT increased significantly and equally in both GT11 and GXB9 on days 30 and 60 after planting. The activity of CAT in GT11 increased gradually until day 60, whereas a similar trend was observed in CA1-inoculated GXB9 until day 30 following planting (Fig. 8a). The PAL expression level showed a significant increase in GT11 on day 60, whereas in GXB9 the increase was observed only on day 30. There was no significant change in the level of PAL expression in GT11 at 30 days and GXB9 at 60 days (Fig. 8b). SOD expression was gradually increased up to 60 days in GT11 for both CY5 and CA1 inoculated strains, whereas, decreased from 30 to 60 days in GXB9 for both inoculated strains (Fig. 8c).

The expression of CHI in GT11 was increased throughout 60 days after planting, while its activity increased for the first 30 days in GXB9 (Fig. 8d). For GT11 and GXB9, the GLU expression increased continuously until day 60, except in CY5-inoculated GXB9 (Fig. 8e).

### Percent of ^15^N_2_ and N content in sugarcane

**Fig. 9:**
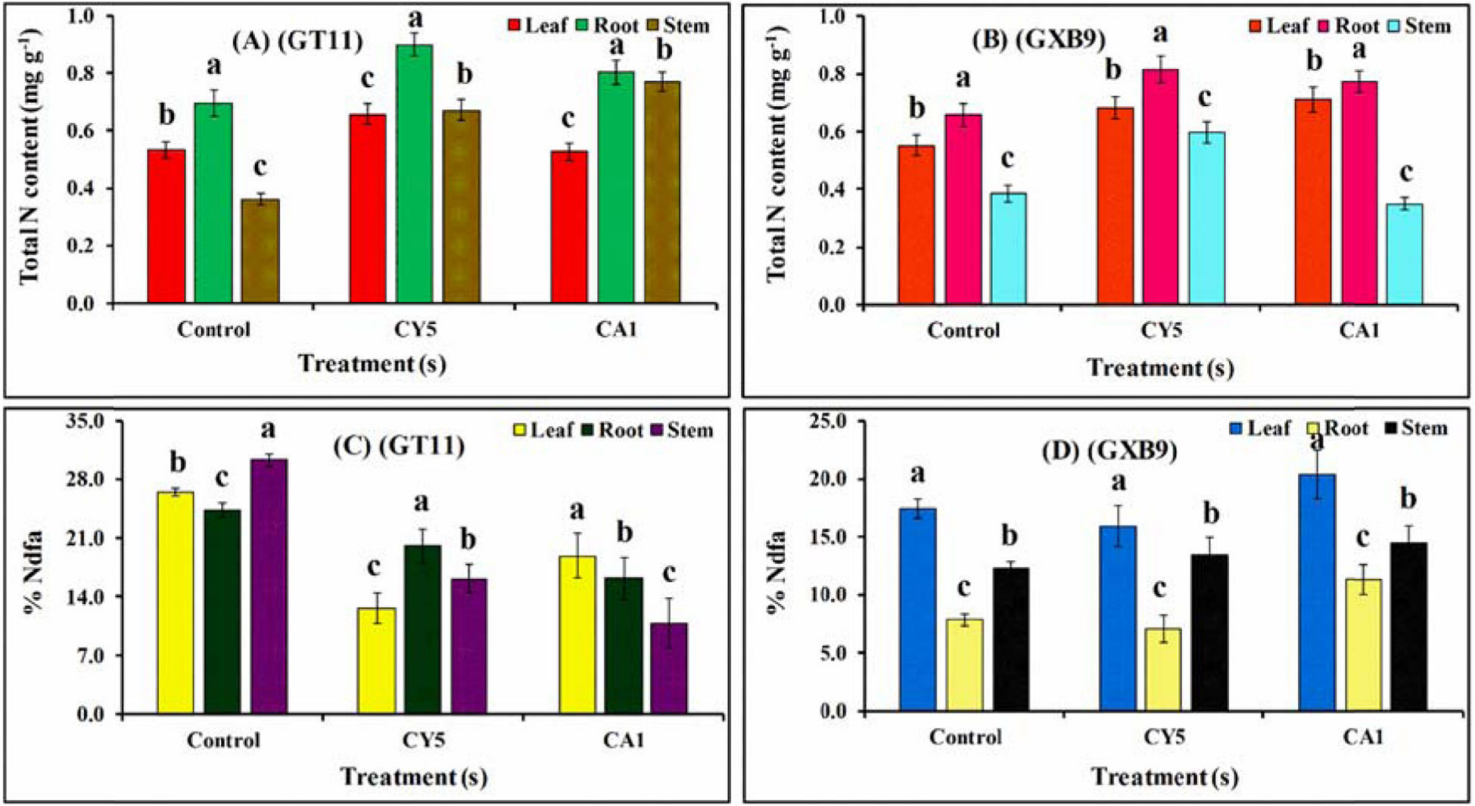
Effect of inoculation of strains *Bacillus megaterium* (CY5) and *Bacillus mycoides* (CA1) on percent ^15^N and N parameters for dry biomass of two sugarcane varieties (GT11 and GXB9). The columns represent the mean of the data for each treatment and bars represent the standard error

The concentration of N content in plant tissues such as leaf, root, and stem was significantly increased with the inoculation of selected isolates (CY5 and CA1) in both sugarcane varieties (GT11 and GXB9) as compared with the control (uninoculated). For GT11, the maximum increase in total N concentration was observed in root samples for plants inoculated with both isolates compared with the control (Fig. 9a). Whereas for GXB9, compared with the control, CY5 inoculation results in more N concentration than that with CA1 in all samples (Fig. 9b).

To estimate the proportion of N in sugarcane plant parts derived from the atmosphere, the plant growth medium was equally tagged with ten mg of H_4_SO_4_ per kilogram of soil. Our results showed that strain CY5 fixed more N in root and stem tissues whereas, less N in leaf tissues of GT11 variety as compared to CA1 strain. However, in the case of GXB9 variety, CA1 fixed more N in all plant parts (leaf, stem, and root) than strain CY5 (Fig. 9c-d). Consequently, the availability of N in inoculated plants allowed improved growth and development in soil-grown plants.

## Discussion

An important objective of research on rhizosphere microbes that fix nitrogen is to extend biological nitrogen fixation as a significant source of nitrogen for non-leguminous crops. It is important to identify a nitrogen-fixing micro-organism in a major agricultural crop like sugarcane. In addition, BNF microorganisms reduce the cost of sugarcane production [5]. In Brazil sugarcane is cultivated with a very low amount of N inputs suggesting the occurrence of BNF [25]. In this work, the evidence of BNF in *B. megaterium* (CY5), and *B. mycoides* (CA1) is well established. Ambrosini et al. [48] previously studied *Bacillus mycoides* B38 V, which produces various PGP traits and biocontrol activity against *Sclerotinia scleratiorum* isolated from sunflower. Here, we confirmed that *Bacillus mycoides* is the nitrogen-fixing as well as plant growth-promoting bacteria in sugarcane. Members of some *Bacillus* spp. showed PGP traits and N-fixing ability, directly affects the plant growth and development [49, 50].

Soil micro-organisms are essential for biogeochemical cycles, colonizing the plant root, improving soil fertility and plant health and increasing crop production [51, 52]. In this specific study, we focused on *Bacillus* genus isolated from the sugarcane rhizosphere and characterized them for PGP traits, in vitro antifungal activities as well as nitrogen-fixing activity. A total of 22 *Bacillus* isolates were identified following16S rRNA and *rpoB* gene-based phylogenetic analysis. All the 22 strains isolated in the present study were selected on the basis of PGP, nitrogenase, biocontrol of pathogens, etc. The strains *Bacillus megaterium* (CY5) and *Bacillus mycoides* (CA1) were the most promising. Therefore, the antagonistic efficacy test can be used as a regular test for screening biocontrol agents and shows a cumulative result of all mechanisms for biocontrol [7, 29]. Nitrogen-fixing bacterial genera especially *Bacillus, Pseudomonas,* and *Enterobacter*, are known to solubilize phosphate compounds present in the soil. Among the 22 bacterial isolates, 18 (81.8%) isolates displayed to phosphate solubilization property by forming clear zones in the plates [7, 30]. Another PGP trait of *Bacillus* species was the activity of siderophore, and it has been recommended that siderophores are involved in plant protection [53, 54]. The qualitative test results showed 59.1 and 18.2% of *Bacillus* strains had ammonium and HCN production ability. Also, all selected screened strains played an important role in biocontrol of *S. scitamineum* and *C. paradoxa* pathogens.

Smut, caused by *S. scitamineum* is a major disease of sugarcane [55], which causes significant yield loss of sugarcane production in Guangxi, China. The average smut infection rate is over 10% and might reach over 50% in some regions in China [55]. Pineapple disease is another common disease in all sugarcane growing regions in China, caused by *C. paradoxa*. Therefore, preventing these diseases, by non-chemical means is now a research priority in China. *Bacillus* strains produced ammonia when grown in nitrogen sources and ammonia accumulating strains supply nitrogen to the host plant and support plant biomass production [56].

The method to examine the quantitative amount of PGPR associated with sugarcane is culture-dependent. A number of bacterial strains expressing ACC deaminase have been isolated in laboratory conditions worldwide and reported their PGPR activity [57, 58]. Many PGPR bacteria stimulate plant growth through the activity of ACC deaminase by reducing plant ethylene levels. Nascimento et al. [59] reported that the ACC deaminase shows various roles in microorganism’s developmental processes along with plant growth promotion abilities and also proposed the ACC deaminase belongs to an extensive group of pyridoxal phosphate-dependent enzymes based on protein sequence and phylogenetic analysis. And, the best ACC-utilizing bacterial isolates are from the genus *Burkholderia* and *Pseudomonas* isolated from sugarcane [60, 61]. Quantitatively, only ten (45.45%) studied strains showed significant ACC deaminase activity, with CY5 and CA1 recording the highest activity. The bacterial strains producing IAA might increase root growth and help develop lateral roots which would increase nutrient uptake from the rhizosphere [62]. We found that all the *Bacillus* strains were able to produce IAA in the range from 35.01 to 130.96 μg mL^− 1^. Also, all the selected strains showed nitrogenase activity and they were confirmed as nitrogen-fixing bacteria, similar to what was observed previously in. *Bacillus mycoides* B38 V, and *Bacillus megaterium* [30, 48].

In this study, we have also examined a molecular method for examining the nitrogen-fixing genes in all the selected strains. For the amplification of the *nifH* gene, several primers were used based on its nucleotide sequences, yet the *nifH* gene was amplified in only six strains. It has been reported that the amplification of the *nifH* gene is useful for confirming the potential strains showing nitrogen fixation [63]. The lack of *nifH* gene amplification does not imply that the isolates are not capable of BNF since the nucleotide sequences of the *nifH* gene in some *Bacillus* species may be significantly different from others [64]. Genomic diversity of *Bacillus* strains in this study showed considerable similarity to those reported earlier [7]. Genetic diversity studies of bacterial species isolated from sugarcane using PCR-based methods are limited. Versalovic et al. [65] described a method of bacterial fingerprinting to examine the strain-specific banding patterns for PCR amplification of repetitive DNA elements presented in entire bacterial genomes. The dendrogram obtained from the cluster analysis of BOX and ERIC-PCR fingerprints provided a complete classification of the species exhibiting biological nitrogen fixation.

Out of all the screened strains, two strains (CY5 and CA1) were characterized using BIOLOG^(R)^ phenotype microarray assays, localization studies using GFP marker, *nifH* gene expression using qRT-PCR and ^15^N_2_ isotope dilution assay in sugarcane plants inoculated with CY5 and CA1. The pattern of metabolic profiling of CY5 and CA1 showed that they use a variety of metabolic substrates (Fig. 4). Wielbo et al. [66] proposed that strains with wide metabolite tolerance were more effective players in host plant nodulation. In this study, the strain CA1 was more metabolically diverse than CY5, suggested that CA1 might be more effective in terms of plant growth and development. The metabolic properties of an organism might play a role in survival to establish successful host assemblage and to promote plant growth and establishment [67].

To examine the effect of bacterial strains on sugarcane colonization capability CY5 and CA1 were genetically tagged with GFP and the amount of colonization was observed by using CLSM. The uninoculated plants after 72 h of incubation showed no fluorescent bacterial colony, whereas, in the plants inoculated with the isolates, it was observed that bacterial cell density had increased, and fluorescent cells were detected and all over the plant organs, including roots, stems, and leaves. Both strains colonized the sugarcane after inoculated independently. Fluorescence due to the GFP-tagged bacterial colonization was detected in all the sugarcane plant parts. Similarly, in the sugarcane plant, fluorescent cells of GFP tagging can be observed on sugarcane roots and leaves but the micro-organisms and their pattern of colonization were different [7, 68].

We determined the amount of *nifH* gene expression for selected strains in sugarcane by qRT-PCR. The *nifH* gene expression through mRNA of diazotrophs indicated the level of biological N_2_ fixation activity, and our results showed both CY5 and CA1 strains, as well as control, expressed nitrogen fixation activity in both sugarcane varieties at different levels. The qRT-PCR method is highly sensitive and specific [69] and suitable for the detection of mRNA transcriptions of micro-organisms at low densities in different plant tissues or experimental samples. The expression profile of important plant development and defense-related enzymes, specifically CAT, PAL, SOD, CHI and GLU was compared by RT-qPCR technique in both sugarcane plants at 30 and 60 days of post-inoculation with CY5 and CA1 strains. Similar to the earlier reports a positive response of antioxidant enzymes (CAT, SOD, and PAL) at different developmental stages in sugarcane against smut disease resistance was observed [70]. The expression of several catalase genes is regulated in response to numerous environmental oxidative stimuli in sugarcane [71] and different developmental stages in maize [72, 73]. Additionally, the positive response of SOD and PAL supports the contention that *Bacillus* species such as CY5 and CA1 help sugarcane tolerate various oxidative stresses [74, 75]. Plant beta-1,3-glucanase and chitinases are the PR proteins, extensively distributed in higher plant roots, stems, and flowers [76–87], and the expression of these proteins has been controlled mostly during stress conditions such as pathogen infection [78]. The expression of beta-1,3-glucanase and chitinase genes has been reported in various physiological and developmental processes of plants [79], like seed and pollen germination [80], flower development and fruit ripening [81].

^15^N_2_ isotope dilution and N balance trials with different cultivars of sugarcane specify their ability to obtain substantial quantities of nitrogen from atmospheric N_2_ through biological nitrogen-fixing micro-organisms [82]. In this study, our results of ^15^N_2_ isotope dilution method and N_2_ assimilation experiments proposed a similar role of biological nitrogen fixation by micro-organisms in sugarcane plants.

## Conclusions

In summary, the present study indicates the occurrence of various strains of *Bacillus* genus as plant-growth promoting and nitrogen-fixing bacteria in the sugarcane rhizosphere. The use of effective nitrogen-fixing micro-organisms is an opportunity for improving crop production in addition to maintaining soil structure and fertility. And, all those isolated bacteria showed various PGP traits, nitrogenase activity, and disease resistance in response to different pathogens. Both strains, *B. megaterium* (CY5), and *B. mycoides* (CA1) may play an important role in sugarcane crop protection, adaptation to environmental stresses and provision of nutrients.

## Methods

### Descriptions of the study site, and soil physico-chemical analysis

The aim of soil testing is to determine the nutrient of the study site. The study site is located in an experimental field of Guangxi University, Nanning, China at latitude 22°49′1.21″ N, longitude 108°21′59.55″ E and an elevation between 70 and 500 m above sea-level. It has a warm, humid subtropical climate with an annual average temperature of 21.7 °C (21–33 °C), and rainfall varied between 1000 mm to 2800. Soil samples were randomly collected from the sugarcane field in April 2015 and stored at 4 °C. The crushed soil samples were analyzed for physical and chemical properties. The electrical conductivity (EC) of rhizosphere soil ranged from 0.00871 to 0.0111 Sm^− 1^, and it depends on the amount of moisture and the size, and texture of soil particles. The water content was varied from 5.13 to 6.18%, and the pH ranged from 6.0 to 6.70. The amount nitrogen, phosphorus, and potassium in the test soil were 0.60, 0.46 and 14.26 g Kg^− 1^ respectively. The level of other macro and micronutrients in the sugarcane rhizosphere are presented in Additional file 5 (Table S2).

### Isolation of nitrogen-fixing bacteria

Five, six months old healthy cultivated sugarcane plants were randomly collected from our experimental field of Sugarcane Research Institute, Guangxi Academy of Agricultural Sciences, Nanning, China. No permissions were required for the collection of sugarcane plant samples in this study. Root-adhered soil was collected and mixed well and then stored at 4 °C for further analysis. Root debris was removed by sieving the soil through a 2 mm mesh. Ten grams of soil sample was suspended in 90 mL of saline water and agitated in an orbital shaker set at 100 rpm and 30 ± 2 °C for 30 min. Different bacterial species present in the soil suspension were isolated as described earlier [29]. Nitrogen-fixing bacteria were isolated by using five different media JNFb medium, LGI Medium [83, 84], Ashbey medium (Hi-Media), Yeast Mannitol Agar (Hi-Media), and Nutrient Agar (Hi-Media) (Additional file 6: Table S3). The colonies of dissimilar morphotypes appeared in the culture were purified and stored for further studies.

### Identification of isolates with antifungal and plant growth promotion properties

All isolates were tested for their in vitro antifungal activity by dual culture method on PDA: NA (1:1) agar plates with two sugarcane pathogens *S. scitamineum*, and *C. paradoxa*. A disk of 5 mm diameter was cut from an actively growing culture in the PDA plate and it was placed at the center of the PDA: NA plates. The bacterial isolates were grown in nutrient broth at a concentration of 10^6^ cell mL^− 1^ and they were streaked on the plate approximately 3 cm from the center where pathogen disk is located [29, 85]. The cultures were then incubated at 28 ± 2 °C for five days or till the fungal mycelia were fully grown in the control plate. Pathogen without bacterial strain was used as a control. The antifungal activity was assessed by determining the growth inhibition against the test pathogen. The Percentage of inhibition was calculated according to Singh et al. [29]. The isolates showing ≥50% inhibition of mycelial growth was considered as promising biocontrol agents.

The plant growth promotion potential of all the selected isolates was assessed by standard procedures that measure phosphate solubilization [52] and the production of siderophore [86], HCN, [87] and ammonia production [88]. IAA production by the isolates was estimated spectrophotometrically following the method described by Glickmann and Dessaux [89].

### 1-Aminocyclopropane-1-carboxylate deaminase assay

All isolates were screened for ACC deaminase activity by using nitrogen-free Dworkin and Foster (DF) medium [90]. Medium without ACC was used as a negative control, whereas two positive controls, i.e. medium with ammonium sulfate [(NH_4_)_2_SO_4_] (0.2% w/v) and those with ACC (3 mM) were also used, and the cultures were incubated at 30 ± 2 °C for 3**–**5 days. The strains grown on ACC plates were further selected and *acdS* gene amplification was observed with degenerate primers and the conditions were followed by Li et al. [91]. The ACC deaminase activity was quantified according to Honma and Shimomura [92].

### Acetylene reduction assay

Each bacterial isolate was examined for nitrogen-fixing ability through the acetylene reduction assay (ARA) method [93]. All isolates were inoculated in a 25 mL flask comprising 10 mL JNFb (Additional file 6) medium and incubated for 3 days at 30 ± 2 °C. Inside air from the tubes is replaced with 5 mL of acetylene through a syringe and the tubes were incubated for 24 h at 30 ± 2 °C [7]. The column (DB-1701, Agilent, Santa Clara, USA) temperature was set at 80 °C, whereas flame ionization detector and injector temperature were 110 °C, with carrier gas nitrogen at a flow rate of 35 mL min^− 1^. A gas sample from the tube (0.5 mL) was inserted into the GC-17A gas chromatograph (Shimadzu, Kyoto, Japan) and the peak heights were measured and to compute ethylene production in samples. The total protein concentration of each sample tube was estimated by the protein assay kit (Solarbio, Life Sciences, Beijing).

### DNA extraction and partial sequencing of 16S rRNA and *rpoB* genes

A pure culture of bacteria was grown in Luria-Bertani broth for 24–36 h on a rotary incubator maintained at 32 ± 2 °C and 150 rpm. DNA was extracted from 1.5 mL of pure culture using a DNA isolation kit (CWBIO, Beijing-China) according to the manufacturer’s instructions. The quantity and integrity of the extracted DNA were determined by electrophoresis (0.8%) and by spectrophotometry, using Nanophotometer (Pearl, Implen-3780).

To amplify the 16S rRNA and *rpoB* gene, primer pairs PA-F and PH-R for the 16S rRNA gene [96 and rpoB-F and rpoB-R for *rpoB* gene [94] were used (Table 5). The polymerase chain reaction (PCR) was performed for16S rRNA gene with a heated lid included in the initial denaturation at 95 °C for 5 min, 30 cycles of denaturation at 95 °C for 1 min, annealing at 55 °C for 1 min, extension at 72 °C for 1 min and *rpoB* gene 95 °C for 3 min, 35 cycles at 95 °C for 20 s, 55 °C for 30 s, and 72 °C for 1.5 min extension and final extension for both genes at 72 °C for 5 min. All amplified products were purified by PCR purification kit (BioFlux) and sequenced (Sangon Biotech, Shanghai, China).

**Table 5:** PCR primers used for species identification, molecular characterization, and gene expression

Target Gene	Primer Name	Nucleotide Sequence (5′ ------- → 3′)	Product Size (bp)	Reference
16S	PA**-**F PH**-**R	AGAGTTTGATCCTGGCTCAG AAGGAGGTGATCCAGCCGCA	1300–1500	[95]
*rpoB*	RPO-F RPO-R	ATCGAAACGCCTGAAGGTCCAAACAT ACACCCTTGTTACCGTGACGACC	> 1100	[94]
BOX	BOX A1R	CTACGGCAAGGCGACGCTGACG	50–5000	[96]
ERIC	ERIC**-**1 ERIC-2	ATGTAAGCTCCTGGGGATTCAC AAGTAAGTGACTGGGGTGAGCG	50–5000	
**RTq-PCR Primers**				
*NifH*	Pol**-**F Pol**-**R	TGCGAYCC-SAARGCBGACTC ATSGCCATCATYTCRCCGGA	RTq-PCR	[97]
Glyceraldehyde 3-phosphate dehydrogenase	GAPDH-1 GAPDH-2	CTCTGCCCCAAGCAAAGATG TGTTGTGCAGCTAGCATTG	RTq-PCR	[98]
*SuPAL*	PAL-F PAL-R	CTCGAGGAGAACATCAAGAC GTGATGAGCTCCTTCTCG	RTq-PCR	[74]
*SuCAT*	CAT-F CAT-R	CTTGTCTGGAGCACATACACTTGGA TTCTCCGCATAGACCTTGAACTTTG	RTq-PCR	[72]
*SuSOD*	SOD-F SOD-R	TTTGTCCAAGAGGGAGATGG CTTCTCCAGCGGTGACATTT	RTq-PCR	[75]
*ScChi*	ScChi-QF ScChi-QR	ACGGCTACGGCGACAACA GTCCGCTGACCAGATGAAGAG	RTq-PCR	[71]
*ScGluD1*	D-QF D-QR	TGCTACTTCTTATCCACCCTCTG CGTTGACATAGAAAGGTGAGCC	RTq-PCR	[99]

### Phylogenetic analysis

To verify the identities of the bacterial isolates and determine their evolutionary relationships, the 16S rRNA and *rpoB* gene sequences with essential reference sequences were sourced from the NCBI GenBank database. The multiple sequences were aligned by ClustalW [100] and the BlastN search program was performed to compare the sequences. The 16S rRNA and *rpoB* genes were subjected to phylogenetic analysis using molecular evolutionary genetics analysis (MEGA) software (version 7.0) [101], and the unweighted pair group method with arithmetic mean (UPGMA) in a Kimura two-parameter model [102]. The bootstrap analysis was carried out by the Felsenstein method using 1000 pseudoreplication [103].

### Amplification, cloning, and sequencing of the *nifH* gene

Using total DNA as a template, the *nifH* gene was amplified by polymerase chain reaction (PCR) using primers PolF and PolR ([97]; Table 5). The PCR was performed as described earlier [7]. The PCR product was gel extracted, purified and cloned (pMD**®** 18-TVector) following the manufacturer’s (TaKaRa, Japan) instructions. The recombinant colonies grown on Luria-Bertani agar plates containing 50 μg mL^− 1^ ampicillin were identified by colony PCR. The cloned PCR products were sequenced (Sangon Biotech, Shanghai, China) to establish the *nifH* gene identity.

### Genetic characterization of isolates by BOX and ERIC-PCR

To determine the genetic diversity of selected isolates, total DNA isolated were fingerprinted by BOX-PCR and ERIC-PCR [29] using the primers and the conditions listed in Table 5, and Additional file 7 (Table S4), respectively. The amplified bands were separated by gel electrophoresis (1.5% agarose) and the gels were imaged by the BIORAD gel documentation system.

### Phenotype microarray assays

Among all the screened isolates CY5 and CA1 were the most potent N-fixing strains on the basis of PGPs as well as nitrogenase activity. Thus, both were selected for further experiments. Phenotype microarray was conducted using Biolog microplates, a tetrazolium-based growth assay developed by Biolog Incorporated (Biolog, Inc., Hayward, CA). The potential strains CY5 and CA1 were assayed on microplates GENIII, PM3B, PM9 and PM10, testing for different substrates such as several carbon and nitrogen sources, and tolerance to different osmotic and pH stresses [104]. The inoculum of microplates was prepared as described [7]. All the microplates were incubated for 72 h at 30 ± 2 °C for tetrazolium color development. After incubation, the readings were obtained using an automated BIOLOG^(R)^ Micro-Station Reader according to the manufacturer’s instructions. The microbial growth was evaluated using optical density measurements at 590 nm after 72 h incubation.

### Genetic transformation of strains with green fluorescent protein

#### Plasmid transformation

The pPROBE-pTet^r^-TT plasmid containing the green fluorescent protein (GFP) gene and tetracycline resistance gene (provided by State Key Laboratory of Subtropical Bioresources Conservation and Utilization, Guangxi University, Nanning, China) was transferred to *Bacillus* strains (CY5 and CA1) by biparental mating using *Escherichia coli* strain TG1 as the donor and DH5α/pRK2013 as the helper [105]. Transformants were selected using kanamycin (100 μg mL^− 1^) and ampicillin (100 μg mL^− 1^) resistance and green fluorescence.

#### Colonization of sugarcane plantlets by GFP-tagged pPROBE-pTet^r^-TT CY5 and CA1 bacteria

Plantlets of micro-propagated cultivated sugarcane variety GT11 were obtained from Sugarcane Research Institute, Guangxi Academy of Agriculture Sciences, Nanning, China, and plantlets were inoculated with *Bacillus* strains and GFP /pPROBEpTet^r^-TT tagged *Bacillus* strains according to Oliveira et al. [105]. Five different plantlets were shifted in a glass container comprising 50 mL of liquid MS medium [106]. After two-three days, plantlets were shifted in bacterial suspension approximately 2.0 × 10^5^ mL^− 1^ cell count in autoclaved bottles and plantlets without bacterial suspension were used as a control, following the growth chamber condition of Li et al. [7].

#### Confocal laser scanning microscopy

After 96 h of bacterial inoculation, inoculated and un-inoculated sugarcane plantlets were removed from the culture vessel and washed two to three times with autoclaved distilled water. The root, stem, and leaf were cut into small parts (50 to 150 μm) and then mounted them on the bridge slide with 10% (v/v) glycerol. All plant sections were detected with a CLSM (Leica DMI 6000, Leica Microsystems, Mannheim, Germany).

### Evaluation of isolates in the greenhouse experiment

The experiment was conducted in a greenhouse with two sugarcane verities GT11 and GXB9, at Guangxi University, Nanning, Guangxi, China. The plantlets of these varieties were provided by Sugarcane Research Institute, Guangxi Academy of Agricultural Sciences, Nanning, China. Forty healthy sugarcane plantlets of each sugarcane variety were divided into two groups i.e., 20–20. These selected plantlets were 30–40 days old, then washed with running tap water to remove all soil particles attached to the surface of the plants. All sugarcane plantlets were immersed in bacterial spore suspension set at 10^6^ spores mL^− 1^ for 1 h. After, these treatments, plantlets were transferred into plastic pots (30 cm in diameter, 40 cm in-depth, two plants per pot) with 15 kg soil and sand mixture (3:1 w/w). The plantlets without spore suspension treatment were used as the control for both varieties. After inoculation, the plantlets were grown in the pots under controlled conditions (30 ± 2 °C, > 80% relative humidity and 16/8 h light/dark cycle). Leaf, stem and root samples from both varieties were sampled at consecutive time intervals (30–120 days). Collected samples were immediately stored at − 80 °C until they were used for investigations.

### RNA extraction and cDNA synthesis

RNA was extracted using Trizol reagent (Tiangen, China) and purified using a RNeasy Plant Mini Kit (Qiagen). The first-strand cDNA from DNase-treated RNA was synthesized using Prime-Script™ RT Reagent Kit (TaKaRa, China) according to the manufacturer’s instructions.

### Real-time PCR quantification of CY5 and CA1

The expression of *nifH,* CAT, PAL, SOD, CHI and GLU genes in leaf tissues of sugarcane during plant-microorganism interaction was analyzed under a greenhouse condition after inoculation of selected strains (CY5 and CA1) in GT11 and GXB9 sugarcane varieties. Both sugarcane varieties were provided by Sugarcane Research Institute, Guangxi Academy of Agricultural Sciences, Nanning, China. The relative expression of all genes was measured by calculating the difference in the expression level of the inoculated sample and the control at 60 and 120 days with glyceraldehyde-3-phosphate dehydrogenase (GAPDH) as the reference gene.

Real-time quantitative PCR **(**RT-qPCR) was conducted with SYBR Premix Ex Tap™II (TaKaRa, Japan) and the reaction was completed in RT-PCR System (Bio-Rad, USA). The total reaction volume was 20 μL, the composition of the reaction mixtures and conditions were followed by Niu et al. [98]. The list of primers used is presented in Table 5. The specificity of the reaction was confirmed by melting curve analysis at the end of the amplification and 2^−△△Ct^ method was used for quantification of relative gene expression [107]. Each RT-qPCR experiment was conducted in triplicate.

### Plant tissue analysis for ^15^N_2_ and %N by isotope dilution assay

The nitrogen content of the dried plant tissues (root, leaf, and stem) was determined by the Institute of Genetics and Physiology, Hebei Academy of Agriculture and Forestry Sciences, China. Atom % ^15^N_2_ was determined by K05 automatic Kjeldahl nitrogen determination apparatus (Shanghai Sonnen automated analysis instrument co. Ltd.), and elementary analysis isotope ratio mass spectrometers (Thermo Fisher DeltaV Advantage IRMS). The ^15^N_2_ isotope dilution assay was used to quantify the nitrogen fixed by CY5 and CA1, in the sugarcane varieties GT11 and GXB9. ^15^N_2_ isotope dilution assay of GT11 and GXB9 plants under the greenhouse condition was done as described by Lin et al. [108].

The method involves testing of an N_2_-fixing crop grown in a substrate with homogenous ^15^N_2_ enrichment ammonium sulfate (10 mg kg^− 1^ of soil). Pots were filled with 15 kg of soil mixed with ^15^N_2_-labeled ammonium sulfate. Three plants of similar sizes and inoculated with CY5 and CA1 strains were transplanted in each pot. There were five replicates per treatment. Six months after planting, the plants were harvested and washed to remove the attached soil. Root, leaf, and stem were separated and dried at 65 °C for 48 h and ground to make a fine powder, then sieved using a 0.5 mm mesh. The percentage of fixed N_2_ derived from the air (Ndfa) in the plant was determined according to the following equation [108].

$$ \%\mathrm{Ndfa}=100\times \left\{1-\left(\mathrm{atom}\%{\;}^{15}{\mathrm{N}}_2\;\mathrm{excess}\kern0.17em \mathrm{from}\kern0.17em \mathrm{treatment}\right)/\left(\mathrm{atom}\%{\;}^{15}{\mathrm{N}}_2\;\mathrm{excess}\kern0.17em \mathrm{from}\kern0.17em \mathrm{control}\right)\right\} $$

### Statistical data analysis

The data were analyzed using analysis of variance (ANOVA) followed by Duncan’s multiple range test (DMRT). Standard errors were calculated for all mean values of three replicates and variations were measured significant at the *p* ≤ 0.05 level by student’s *t*-test. All plant growth-promoting rhizobacteria tests were conducted in triplicate, and the results were expressed as mean values. All the experiments including Biolog, microbial colonization, and RT-PCR were performed in duplicate and the results were assessed for consistency. OrigiPro 9.1 (2013) software was used for the principal component analysis (PCA).

## Supplementary information

**Additional files 1: Figure S1.***acds* gene amplification in nitrogen-fixing bacteria and approximately 755 bp fragments to be amplified. M is a molecular size marker (100 to 2000 bp), PC is positive control (*Pseudomonas entomophila*), and NC is negative control (sterile water).

**Additional files 2: Figure S2.** PCR amplification with genomic DNA of nitrogen-fixing bacteria. The *nifH* gene fragment is amplified at 360 bp. M is a molecular size marker (100 to 2000 bp), *Klebsiella verticola* as a positive control (PC), and sterile water is negative control (NC).

**Additional files 3: Figure S3.** BOX fingerprinting of the screened bacterial isolates from sugarcane. Strain Codes: B1. CoY3, B2. CoY7, B3. CoY8, B4. CoA1, B5. CoA10, B6. AY6, B7. AY7, B8. AY8, B9. AN8, B10. AN11, B11. AN12, B12. BN5, B13. CY5, B14. CY9, B15. CY10, B16. CY11, B17. CA1, B18. CA6, B19. CA8, B20. CN13, B21. CN14 and B22. N1. M, molecular size marker from 100 bp- 5 kb (Takara). **Figure S4.** ERIC-PCR fingerprinting of selected bacteria from sugarcane. Strain Codes: B1. CoY3, B2. CoY7, B3. CoY8, B4. CoA1, B5. CoA10, B6. AY6, B7. AY7, B8. AY8, B9. AN8, B10. AN11, B11. AN12, B12. BN5, B13. CY5, B14. CY9, B15. CY10, B16. CY11, B17. CA1, B18. CA6, B19. CA8, B20. CN13, B21. CN14 and B22. N1. Without labelled gel lanes are not used in this study.

**Additional files 4: Table S1.** The number of substrates utilized by individual strains for *Bacillus megaterium* (CY5) and *Bacillus mycoides* (CA1).

**Additional files 5: Table S2.** Analysis of physical, chemical properties and trace elements structure in the soil of sugarcane plant.

**Additional files 6: Table S3.** The media used in this study for isolation of N_2_ fixing bacteria from sugarcane.

**Additional files 7: Table S4.** Composition and conditions of PCR amplification used for genetic fingerprinting analysis.

## Data Availability

All data produced or evaluated through this study will be easily accessible with request to corresponding authors for reasonable request.

## Abbreviations

ACC: 1-aminocyclopropane-1-carboxylate
ANOVA: Analysis of variance
ARA: Acetylene reduction assay
BLAST: *Basic local alignment search tool*
BOX: A1R-based repetitive extragenic palindromic
CAT: Catalase
CHI: Chitinase
DMRT: Duncan’s multiple range test
EC: Electrical conductivity
ERIC: Enterobacterial repetitive intergenic consensus
GFP: Green fluorescent protein
GLU: Glucanase
HCN: Hydrogen cyanide
IAA: Indole acetic acid
MEGA: Molecular evolutionary genetics analysis
N: Nitrogen
(NH_4_)_2_SO_4_: Ammonium sulfate
NCBI: National center for biotechnology information
PGPR: Plant growth-promoting rhizobacteria
PAL: Phenylalanine ammonia-lyase
PCA: Principal component analysis
PCR: Polymerase chain reaction
qRT-PCR: Quantitative Real-Time polymerase chain reaction
SOD: Superoxide dismutase
UPGMA: Unweighted pair group method with arithmetic mean
