# Supplementary material for: Diversity of nitrogen-fixing rhizobacteria associated with sugarcane: a comprehensive study of plant-microbe interactions for growth enhancement in Saccharum spp
Source: BMC Plant Biol. 2020 May 18;20:220. doi: 10.1186/s12870-020-02400-9 (PMC7236179; doi:10.1186/s12870-020-02400-9)
Supplement: Supplementary file 1 — Additional files 1: Figure S1.acds gene amplification in nitrogen-fixing bacteria and approximately 755 bp fragments to be amplified. M is a molecular size marker (100 to 2000 bp), PC is positive control (Pseudomonas entomophila), and NC is negative control (sterile water). [file 12870_2020_2400_MOESM1_ESM.docx]

**Figure S1.** *acds* gene amplification in nitrogen-fixing bacteria and approximately 755 bp fragments to be amplified. M is a molecular size marker (100 to 2,000 bp), PC is positive control (*Pseudomonas entomophila*), and NC is negative control (sterile water).

| **Target Gene** | **Primer**  **Name** | **Nucleotide Sequence (5**′ **-------→ 3′)** | **Product Size (bp)** | **Reference** |
| --- | --- | --- | --- | --- |
| *acdS* | ACD-F  ACD-R | GCAACAAGACGCGCAAGYTNGARTAYN T  GTGCATCGACTTGCCCTCRWANACNGG RT | 755 | [ Li. 2011] |

**Reference:**

Li ZY. High-throughput screening and identiﬁcation of bacteria containing acc deaminase. Doctoral dissertation, Zhejiang University. 2011, Hangzhou.
