# Supplementary material for: Diversity of nitrogen-fixing rhizobacteria associated with sugarcane: a comprehensive study of plant-microbe interactions for growth enhancement in Saccharum spp
Source: BMC Plant Biol. 2020 May 18;20:220. doi: 10.1186/s12870-020-02400-9 (PMC7236179; doi:10.1186/s12870-020-02400-9)
Supplement: Supplementary file 2 — Additional files 2: Figure S2. PCR amplification with genomic DNA of nitrogen-fixing bacteria. The nifH gene fragment is amplified at 360 bp. M is a molecular size marker (100 to 2000 bp), Klebsiella verticola as a positive control (PC), and sterile water is negative control (NC). [file 12870_2020_2400_MOESM2_ESM.docx]

**Figure S2.** PCR amplification with genomic DNA of nitrogen-fixing bacteria. The *nifH* gene fragment is ampliﬁed at 360 bp. M is a molecular size marker (100 to 2,000 bp), *Klebsiella verticola* as a positive control (PC), and sterile water is negative control (NC).

| **Target Gene** | **Primer**  **Name** | **Nucleotide Sequence (5**′ **-------→ 3′)** | **Product Size (bp)** | **Reference** |
| --- | --- | --- | --- | --- |
| *nifH* | Pol**-**F  Pol**-**R | TGCGAYCCSAARGCBGACTC  ATSGCCATCATYTCRCCGGA | 360 | [[Poly](http://www.sciencedirect.com/science/article/pii/S0923250800011724) et al. 2001] |

**Reference:**

[Poly](http://www.sciencedirect.com/science/article/pii/S0923250800011724) F, [Monrozier](http://www.sciencedirect.com/science/article/pii/S0923250800011724) LJ, [Bally](http://www.sciencedirect.com/science/article/pii/S0923250800011724) R. Improvement in the RFLP procedure for studying the diversity of *nifH* genes in communities of nitrogen fixers in soil. Res Microbiol. 2001; 152:95-103.
