# Supplementary material for: Diversity of nitrogen-fixing rhizobacteria associated with sugarcane: a comprehensive study of plant-microbe interactions for growth enhancement in Saccharum spp
Source: BMC Plant Biol. 2020 May 18;20:220. doi: 10.1186/s12870-020-02400-9 (PMC7236179; doi:10.1186/s12870-020-02400-9)
Supplement: Supplementary file 3 — Additional files 3: Figure S3. BOX fingerprinting of the screened bacterial isolates from sugarcane. Strain Codes: B1. CoY3, B2. CoY7, B3. CoY8, B4. CoA1, B5. CoA10, B6. AY6, B7. AY7, B8. AY8, B9. AN8, B10. AN11, B11. AN12, B12. BN5, B13. CY5, B14. CY9, B15. CY10, B16. CY11, B17. CA1, B18. CA6, B19. CA8, B20. CN13, B21. CN14 and B22. N1. M, molecular size marker from 100 bp- 5 kb (Takara). Figure S4. ERIC-PCR fingerprinting of selected bacteria from sugarcane. Strain Codes: B1. CoY3, B2. CoY7, B3. CoY8, B4. CoA1, B5. CoA10, B6. AY6, B7. AY7, B8. AY8, B9. AN8, B10. AN11, B11. AN12, B12. BN5, B13. CY5, B14. CY9, B15. CY10, B16. CY11, B17. CA1, B18. CA6, B19. CA8, B20. CN13, B21. CN14 and B22. N1. Without labelled gel lanes are not used in this study. [file 12870_2020_2400_MOESM3_ESM.docx]

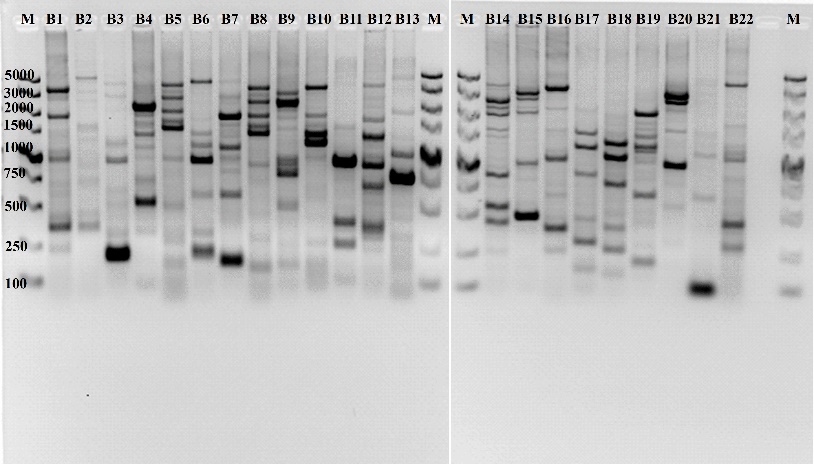


**Figure S3.** BOX fingerprinting of the screened bacterial isolates from sugarcane.

Strain Codes: B1. CoY3, B2. CoY7, B3. CoY8, B4. CoA1, B5. CoA10, B6. AY6, B7. AY7, B8. AY8, B9. AN8, B10. AN11, B11. AN12, B12. BN5, B13. CY5, B14. CY9, B15. CY10, B16. CY11, B17. CA1, B18. CA6, B19. CA8, B20. CN13, B21. CN14 and B22. N1. M, molecular size marker from 100 bp- 5 kb (Takara).


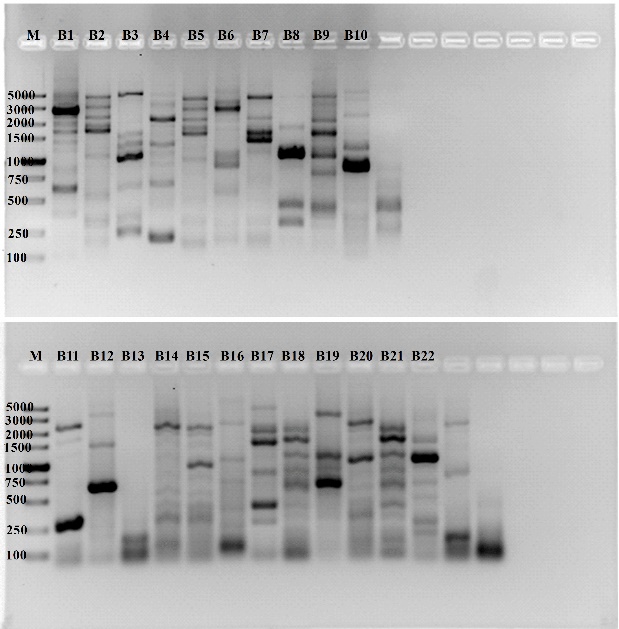


**Figure S4.** ERIC-PCR fingerprinting of selected bacteria from sugarcane.

Strain Codes: B1. CoY3, B2. CoY7, B3. CoY8, B4. CoA1, B5. CoA10, B6. AY6, B7. AY7, B8. AY8, B9. AN8, B10. AN11, B11. AN12, B12. BN5, B13. CY5, B14. CY9, B15. CY10, B16. CY11, B17. CA1, B18. CA6, B19. CA8, B20. CN13, B21. CN14 and B22. N1. Without labelled gel lanes are not used in this study.
