# Supplementary material for: Diversity of nitrogen-fixing rhizobacteria associated with sugarcane: a comprehensive study of plant-microbe interactions for growth enhancement in Saccharum spp
Source: BMC Plant Biol. 2020 May 18;20:220. doi: 10.1186/s12870-020-02400-9 (PMC7236179; doi:10.1186/s12870-020-02400-9)
Supplement: Supplementary file 4 — Additional files 4: Table S1. The number of substrates utilized by individual strains for Bacillus megaterium (CY5) and Bacillus mycoides (CA1). [file 12870_2020_2400_MOESM4_ESM.docx]

**Table S1.** The number of substrates utilized by individual strains for *Bacillus megaterium* (CY5) and *Bacillus mycoides* (CA1).

| **Chemical Guild** | **Total number of Substrate** |  | **CY5** | **CA1** |
| --- | --- | --- | --- | --- |
| Sugars | 27 |  | 15 | 20 |
| Chemical sensitivity | 23 |  | 17 | 20 |
| Acidic pH | 2 |  | 2 | 2 |
| Sodium Chloride | 3 |  | 3 | 3 |
| Lactic acid | 1 |  | 1 | 1 |
| Hexose-PO_4_ | 2 |  | 1 | 2 |
| Amino acid | 9 |  | 6 | 9 |
| Hexose acid | 9 |  | 6 | 8 |
| Reducing Sugar | 2 |  | 2 | 2 |
| Carboxylic acids, esters, and fatty acids | 18 |  | 8 | 12 |
