# Supplementary material for: Diversity of nitrogen-fixing rhizobacteria associated with sugarcane: a comprehensive study of plant-microbe interactions for growth enhancement in Saccharum spp
Source: BMC Plant Biol. 2020 May 18;20:220. doi: 10.1186/s12870-020-02400-9 (PMC7236179; doi:10.1186/s12870-020-02400-9)
Supplement: Supplementary file 5 — Additional files 5: Table S2. Analysis of physical, chemical properties and trace elements structure in the soil of sugarcane plant. [file 12870_2020_2400_MOESM5_ESM.docx]

**Table S2.** Analysis of physical, chemical properties and trace elements structure in the soil of sugarcane plant.

| **Physical properties** | **Analysis Results** |
| --- | --- |
| Soil color | Pale red |
| Soil type | Medium loam |
| Sand (2.0-0.05 mm) (g kg^-1^) | 197.32 |
| Silt (2.0-0.05 mm) (g kg^-1^) | 380.04 |
| Clay (2.0-0.05 mm) (g kg^-1^) | 250.81 |
| EC (Sm^−1^) (minimum to maximum) | 0.00871-0.0111 |
| Water content (%) (minimum to maximum) | 5.13-6.18 |
| pH (minimum to maximum) | 5.99-6.70 |
| **Primary macronutrients (g kg^-1^)** | |
| Total Nitrogen (N) | 0.60 |
| Total Phosphorus (P) | 0.46 |
| Total Potassium (K) | 14.26 |
| **Secondary macronutrients (mg kg^-1^)** | |
| Calcium (Ca) | 813.63 |
| Magnesium (Mg) | 152.46 |
| Sulfur (S) | 144.47 |
| **Micronutrients (mg kg^-1^)** | |
| Iron (Fe) | 150.31 |
| Manganese (Mn) | 89.56 |
| Zinc (Zn) | 8.17 |
| Boron (B) | 0.49 |
| Chlorine (Cl) | 38.49 |
