# Supplementary material for: Diversity of nitrogen-fixing rhizobacteria associated with sugarcane: a comprehensive study of plant-microbe interactions for growth enhancement in Saccharum spp
Source: BMC Plant Biol. 2020 May 18;20:220. doi: 10.1186/s12870-020-02400-9 (PMC7236179; doi:10.1186/s12870-020-02400-9)
Supplement: Supplementary file 6 — Additional files 6: Table S3. The media used in this study for isolation of N2 fixing bacteria from sugarcane. [file 12870_2020_2400_MOESM6_ESM.docx]

**Table S3.** The media used in this study for isolation of N_2_ fixing bacteria from sugarcane.

| **S. No.** | **Name of medium and composition (g L^-1^)** |
| --- | --- |
| 1 | **Ashbey Medium:** Mannitol; 15, CaCl_2_.2H_2_O; 0.2, MgSO_4_.7H_2_O; 0.2, MoO_3_ (10% solution); 0.1 mL, FeCl_3_ (10% solution); 0.05 mL, Agar; 15 |
| 2 | **Yeast Mannitol Agar Medium:** Mannitol; 15, K_2_HPO_4_; 0.5, Yeast Extract; 0.4, MgSO_4_.7H_2_O; 0.2, NaCl; 0.1, Agar; 15 |
| 3 | **LGI Medium:**Sucrose; 5, KH_2_PO_4_; 0.6; K_2_HPO_4_; 0.2; MgSO_4_.7H_2_O; 0.2, CaCl_2_.2H_2_O; 0.02, FeCl_3_; 1, Na_2_MoO_4_. 2H_2_O; 2 mg, Bromothymol Blue Solution; 5 mL, Agar; 1.75 |
| 4 | **Nutrient Agar:** Peptone; 5, NaCl; 5, Yeast Extract; 2, Beef Extract; 1, Agar; 15 |
| 5 | **Dworkin and Foster minimal medium:** Glucose; 2, Gluconic acid; 2, Citric acid; 2, KH_2_PO_4_; 4, Na_2_HPO_4_; 6, MgSO_4_**.**7H_2_O; 0.2, Micro nutrient solution (in mg) (CaCl_2_; 200, FeSO_4_.7H_2_O; 200, H_3_BO_3_; 15, ZnSO_4_.7H_2_O; 20, Na_2_MoO_4_; 10, KI; 10, NaBr; 10, MnCl_2_; 10, COCl_2_; 5, CuCl_2_; 5, AlCl_3_; 2, NiSO_4_; 2. |
| 6 | **JNFb medium:** Malic acid, 5.0; K_2_HPO_4_, 0.6; KH_2_PO_4_, 1.8; MgSO_4_.7H_2_O, 0.2; NaCl, 0.1; CaCl_2_.2H_2_O, 0.02.  Micronutrient solution (CuSO_4_.5H_2_O, 0.04; ZnSO_4_.7H_2_O, 0.12; H_3_BO_3_, 1.40; Na_2_MoO_4_.2H_2_O, 1.0; MnSO_4_. H_2_O, 1.175) (g L^-1^).  2 mL; bromothymol blue (5 g L^−1^ in 0.2 N KOH), 2 mL; FeEDTA (16.4 g L^−1^), 4 mL; vitamin solution (biotin, 10 mg; pyridoxal-HCl, 20 mg, dissolved in hot-water bath. Complete to 100 mL by adding distilled water), 1 mL; KOH, 4.5g. Add distilled water to bring a total solution to 1,000 mL. Adjust the pH to 6.8 with KOH. |
