# Supplementary material for: Diversity of nitrogen-fixing rhizobacteria associated with sugarcane: a comprehensive study of plant-microbe interactions for growth enhancement in Saccharum spp
Source: BMC Plant Biol. 2020 May 18;20:220. doi: 10.1186/s12870-020-02400-9 (PMC7236179; doi:10.1186/s12870-020-02400-9)
Supplement: Supplementary file 7 — Additional files 7: Table S4. Composition and conditions of PCR amplification used for genetic fingerprinting analysis. [file 12870_2020_2400_MOESM7_ESM.docx]

**Table S4.** Composition and conditions of PCR amplification used for genetic fingerprinting analysis.

| **PCR condition** | **Type of PCR** | |
| --- | --- | --- |
| **A. Reaction mixture** | **BOX** | **ERIC** |
| Taq-/Gitschier-buffer | 5 µL | 5 µL |
| BSA (20 mg mL^-1^) | 0.2 µL | 0.2 µL |
| DMSO (100%) | 2.5 µL | 2.5 µL |
| Taq polymerase (3 U) | 0.6 µL | 0.6 µL |
| dNTP mixture (100 mM mix) | 1.25 µL | 1.25 µL |
| Primer (Forward) | 1.3 µL (single primer) | 1.3 µL |
| Primer (Reverse) | **-** | 1.3 µL |
| Template DNA | 1.2 µL | 1.2 µL |
| Water (Molecular grade) | 12.95µL | 11.65 µL |
| **B. Reaction conditions** | | |
| Lead heat | 110°C | 110°C |
| Initial Temperature | 94°C for 3m | 95°C for 4m |
| Start cycles | **-** | **-** |
| Number of cycles | 35 | 35 |
| Denaturation’s | 94°C for 30s | 94°C for 1m |
| Annealing | 50°C for 1m | 52°C for 1m |
| Elongation | 72°C for 8m | 65°C for 8m |
| End cycles | **-** | **-** |
| Final extensions | 72°C for 16m | 65°C for 16m |
